# *Ppp2r2a* Knockout Mice Reveal That Protein Phosphatase 2A Regulatory Subunit, PP2A-B55α, Is an Essential Regulator of Neuronal and Epidermal Embryonic Development

**DOI:** 10.3389/fcell.2020.00358

**Published:** 2020-06-05

**Authors:** Nikita Panicker, Melody Coutman, Charley Lawlor-O’Neill, Richard G. S. Kahl, Séverine Roselli, Nicole M. Verrills

**Affiliations:** ^1^School of Biomedical Sciences and Pharmacy, Faculty of Health and Medicine, Priority Research Centre for Cancer Research, Innovation and Translation, University of Newcastle, Callaghan, NSW, Australia; ^2^Hunter Cancer Research Alliance, Hunter Medical Research Institute, New Lambton, NSW, Australia

**Keywords:** PP2A, knockout mouse, embryo, lethality, skin, epidermal barrier

## Abstract

The serine/threonine protein phosphatase 2A (PP2A) is a master regulator of the complex cellular signaling that occurs during all stages of mammalian development. PP2A is composed of a catalytic, a structural, and regulatory subunit, for which there are multiple isoforms. The association of specific regulatory subunits determines substrate specificity and localization of phosphatase activity, however, the precise role of each regulatory subunit in development is not known. Here we report the generation of the first knockout mouse for the *Ppp2r2a* gene, encoding the PP2A-B55α regulatory subunit, using CRISPR/Cas9. Heterozygous animals developed and grew as normal, however, homozygous knockout mice were not viable. Analysis of embryos at different developmental stages found a normal Mendelian ratio of *Ppp2r2a^–/–^* embryos at embryonic day (E) 10.5 (25%), but reduced *Ppp2r2a^–/–^* embryos at E14.5 (18%), and further reduced at E18.5 (10%). No live *Ppp2r2a^–/–^* pups were observed at birth. *Ppp2r2a^–/–^* embryos were significantly smaller than wild-type or heterozygous littermates and displayed a variety of neural defects such as exencephaly, spina bifida, and cranial vault collapse, as well as syndactyly and severe epidermal defects; all processes driven by growth and differentiation of the ectoderm. *Ppp2r2a^–/–^* embryos had incomplete epidermal barrier acquisition, associated with thin, poorly differentiated stratified epithelium with weak attachment to the underlying dermis. The basal keratinocytes in *Ppp2r2a^–/–^* embryos were highly disorganized, with reduced immunolabeling of integrins and basement membrane proteins, suggesting impaired focal adhesion and hemidesmosome assembly. The spinous and granular layers were thinner in the *Ppp2r2a^–/–^* embryos, with aberrant expression of adherens and tight junction associated proteins. The overlying stratum corneum was either absent or incomplete. Thus PP2A-B55α is an essential regulator of epidermal stratification, and is essential for ectodermal development during embryogenesis.

## Introduction

Mammalian development is a highly complex process requiring exquisite regulation of cellular growth, differentiation and morphogenesis. The signaling pathways controlling these processes are mediated, in large part, by phosphorylation cascades. Protein phosphatases control the rate and duration of these signals, yet our understanding of the functional roles of protein phosphatases in mammalian development is poor. Protein phosphatase 2A (PP2A) is a ubiquitously expressed serine-threonine phosphatase that has key roles in growth, differentiation and morphogenesis (Olsen et al., 2006; Moorhead et al., 2007). Serine and threonine residues dominate the human phosphoproteome, together representing 98% of phosphorylation sites identified. Together with protein phosphatase 1 (PP1), PP2A is responsible for over 90% of Ser/Thr de-phosphorylation in most cells (Eichhorn et al., 2009). Dysregulated PP2A activity is associated with numerous diseases, including, but not limited to, Alzheimer’s disease, various cancers, diabetes, cardiac disease, asthma, inflammation and auto-immune conditions (Calin et al., 2000; Takagi et al., 2000; Suzuki and Takahashi, 2003; Neviani et al., 2005; Kowluru and Matti, 2012; Collison et al., 2013; Crispin et al., 2013; Sontag and Sontag, 2014; Lei et al., 2015; Goldsworthy et al., 2016; Ross et al., 2017; Refaey et al., 2019).

Protein phosphatase 2A is a complex family of enzymes, composed of structural (A), catalytic (C) and regulatory (B) subunits. The PP2A core dimer consists of a structural and catalytic subunit, of which there are two isoforms (α and β) expressed in mammals (Hemmings et al., 1990). The tissue and substrate specificity of PP2A activity is mediated by binding of one of the four families of regulatory B-subunits: B55, B56, B″, and B″′ (each with multiple isoforms) to the core dimers. This results in over 80 different potential PP2A configurations (Smith A.S. et al., 2011), which can target a multitude of largely mutually exclusive substrate proteins (e.g., AKT, ERK, cJun, etc.), in diverse signaling pathways, including DNA damage repair, cell cycle progression and mitosis, proliferation, apoptosis and metabolism. Most regulatory B-subunits are ubiquitously expressed, but some demonstrate tissue specificity. For example, members of the B56 family are highly expressed in almost all major organs and tissues (Reynhout and Janssens, 2019). In contrast, the B″ family generally shows low expression in most tissues, with the exception of high B″α in the heart and high B″γ in embryonic brain. The B55 family is highly expressed in the brain, with B55γ almost exclusively expressed in embryonic and adult brain, and other family members to a lower extent. The B55α subunit shows the widest expression pattern within the B55 family (Reynhout and Janssens, 2019). The specific substrates and the functional roles of each regulatory subunit in health and disease, however, have not been well studied.

*In vivo* studies, using knockout mouse models for genes encoding the catalytic and structural subunits, have revealed a vital role for PP2A in embryonic development [recently reviewed in Reynhout and Janssens (2019)]. Knockout of PP2A-Cα (*Ppp2ca*) causes lethality at embryonic day (E) 6.5 (Götz et al., 1998), and knockout of PP2A-Aα (*Ppp2r1a*) results in lethality before E10.5, implying no functional redundancy with the β isoforms of either the A or C subunits in embryonic development, despite high sequence similarity (Ruediger et al., 2011). Of the regulatory B-subunits, knockout of three members have been reported. In the case of B56δ, there are opposing reports, with one study finding homozygous *Ppp2r5d*^–/^*^–^* animals were viable (Louis et al., 2011), and another that mice heterozygous for a strongly hypomorphic *Ppp2r5d* gene-trap allele were viable, but no homozygotes were recovered (Kapfhamer et al., 2010). Constitutive homozygous deletion of *Ppp2r5c* (B56γ) resulted in neonatal death at post-natal (P) day 1–2 due to heart defects (Varadkar et al., 2014); and gene-trap mediated constitutive *Ppp2r5a* (B56α) knockout mice were viable, however, displayed heart and nerve defects (Little et al., 2015), skin lesions, hyperproliferation of the epidermis and hair follicles, and increased hematopoiesis (Janghorban et al., 2017). Thus, specific PP2A B56 subunits play diverse roles in mammalian development. To date, however, the role of B55 subunits in mammalian development is not known.

The PP2A-B55α subunit (encoded by the *PPP2R2A* gene) is expressed in all tissues, with highest levels observed in the embryonic central nervous system and limbs, as well as adult brain, bladder, adrenal glands, ovaries and placenta (Reynhout and Janssens, 2019). Inactivation or genetic loss of B55α has been implicated in human diseases including cancer and Alzheimer’s disease (Sontag et al., 2004; Ruvolo et al., 2011; Kalev et al., 2012; Beca et al., 2015; Watt et al., 2017). Therefore understanding the functional role of B55α in normal physiology is of particular interest. In cellular models, PP2A-B55α complexes have been shown to exert positive regulation of the ERK/MAPK pathway, but negative regulation of the PI3K/AKT pathway (Smith A.M. et al., 2011). PP2A-AB55αC complexes have also been implicated in DNA damage repair pathways by dephosphorylating ATM; cell cycle regulation by dephosphorylation and activation of the retinoblastoma-related protein p107; control of mitotic exit by deactivation of Cyclin-dependent kinase B-Cdk1 and many of its target proteins (Burgess et al., 2010; Gharbi-Ayachi et al., 2010; Manchado et al., 2010; Mochida et al., 2010; Schmitz et al., 2010; Cundell et al., 2013); and cell adhesion and migration by dephosphorylation of Rac1 and AP-1 (Gilan et al., 2015; Bousquet et al., 2016). PP2A-B55α has emerged as a tumor-suppressor in many epithelial and blood cancers. The *PPP2R2A* gene is commonly deleted in human breast (Curtis et al., 2012) and prostate tumors (Cheng et al., 2011), and *PPP2R2A* knockdown in breast cancer cell lines increases tumorigenicity (Watt et al., 2017). *PPP2R2A* is also commonly down-regulated in non-small cell lung carcinomas (Kalev et al., 2012). A recent ENU-induced mutagenesis study reported a splice-site mutation in *Ppp2r2a* resulting in reduced PP2A-B55α expression. Generation of double heterozygous mice for this *Ppp2r2a* mutation and a null allele of the gene encoding the insulin receptor, resulted in a diabetic phenotype characterized by hyperglycemia, hyperinsulinemia, impaired glucose tolerance, and glycosuria (Goldsworthy et al., 2016), suggesting PP2A-B55α may play a role in metabolism and insulin signaling.

PP2A-B55α dephosphorylates β-catenin during Wnt signaling, suggesting it may play a role in development (Zhang et al., 2009). In support of this, *ex vivo* knockdown of PP2A-B55α suggested an essential role for this subunit in mouse oocyte maturation (Liang et al., 2017) and in epidermal barrier formation (O’Shaughnessy et al., 2009). To investigate the function of this subunit *in vivo*, we have generated the first constitutive *Ppp2r2a* knockout mouse using CRISPR/Cas9. Homozygous *Ppp2r2a* deletion resulted in embryonic lethality post E10.5. *Ppp2r2a^–/–^* embryos displayed neural tube defects, limb defects and impaired epidermal barrier formation. The latter was associated with aberrant polarization of basal keratinocytes and aberrant keratinocyte differentiation. Therefore PP2A-B55α has essential roles in embryonic development, in particular in epidermal barrier formation.

## Materials and Methods

### Generation of *Ppp2r2a* Knockout Mice

*Ppp2r2a* knockout mice were generated at the Australian Phenomics Network (Monash University, Melbourne, Australia), using the CRISPR/Cas9 technology. The CRISPR Design site http://crispr.mit.edu/ was used to identify guide RNA target sites flanking exon ENSMUSE00000482200 (exon 4) of the *Ppp2r2a* gene. The following guide RNAs were used: – 5′ TACGATAAAGCAGCCTAGTT 3′ for the 5′ end of exon 4, and – 5′ TTTGCTTTCAGGTACTACAT 3′ for the 3′ end of exon 4. Complementary oligonucleotides corresponding to the RNA guide target sites were annealed and cloned into *Bbs*I (NEB) digested plasmid pX330-U6-Chimeric_BB-CBh-hSpCas9 (Addgene plasmid #42230). Single guide RNAs (sgRNA) were generated using the HiScribe^TM^ T7 Quick High Yield RNA Synthesis Kit (NEB) according to the manufacturer’s instructions. The sgRNAs were purified using the RNeasy Mini Kit (Qiagen). Cas9 mRNA (30 ng/mL, Sigma) and the sgRNAs (15 ng/mL) were microinjected into the cytoplasm of C57BL/6J zygotes at the pronuclei stage. Injected zygotes were transferred into the uterus of pseudopregnant F1 (C57BL/6 × CBA) females. Forward (5′ GTGTTCCAGCCAGCTGTTTCT 3′) and reverse (5′ GACACTGCTGCCTATGTCTGCT 3′) genotyping PCR primers flanking the targeted region and amplifying a product of 819 bp from the wild-type DNA were used to characterize gene editing events in the resulting mice.

### Animals and Genotyping

Animals were used in accordance with the Australian Code of Practice for the Care and Use of Animals for Scientific Purposes and all protocols were approved by the University of Newcastle Animal Care and Ethics Committee. Mice were housed in individually ventilated cages on a 12 h light/dark cycle and fed standard chow *ad libitum*. Timed matings between heterozygous breeding pairs were set up with midday the next day designated as 0.5 days post coitus (dpc), and pregnant mice identified via the presence of a vaginal plug. Mice were euthanased using CO_2_ asphyxiation, and embryos harvested at 10.5 days of development (E10.5), E14.5 and E18.5. Pregnant females were monitored and the day of birth of the pups was designated P0. Euthanasia of E18.5 embryos and neonates was performed by decapitation. Genotyping was performed by PCR on DNA extracted from tail tips, using the REDExtract-N-Amp^TM^ Tissue PCR Kit (Sigma-Aldrich #XNAT) as per the manufacturer’s recommendations. The same PCR primers flanking exon 4 and used to characterize the gene editing events (described above) were used.

### Protein Extraction and Immunoblotting

Protein extraction was conducted by grinding whole embryos to a fine powder in liquid nitrogen, using a mortar and pestle, and proteins solubilized using RIPA lysis buffer (0.05M Hepes pH 7.4, 1% Triton X-100, 0.1% SDS, 50 mM Sodium Fluoride, 0.05M EDTA, 1 mM Sodium Orthovanadate, 2.5% Protease Inhibitor Cocktail (Sigma #P8340), 5% Sodium Deoxycholate). Protein was quantitated using a BCA assay, separated using 4–12% Bis-Tris SDS-PAGE and transferred to nitrocellulose. Immunoblotting was performed using a commercial anti-PP2A-B55α rabbit polyclonal (Cell Signaling Technology #4953); an in-house rabbit polyclonal antibody raised against the B55α peptide FSQVKGAVDDDVAE (residues 14–27) (Strack et al., 1998); a polyclonal rabbit anti-PP2A-A (Merck Millipore #07-250); a mouse monoclonal anti-PP2A-C (Merck Millipore #05-421); and anti-rabbit/mouse HRP-conjugated secondary antibodies. A HRP-conjugated anti-actin antibody (Sigma #A3854) was used as a loading control. Images were captured on a Biorad ChemiDoc^TM^ Imaging system using ECL.

### RNA Isolation, cDNA Synthesis and qPCR

Total RNA was prepared from embryonic tissue using the Isolate II RNA Mini Kit (Bioline #BIO-52072) and 1 μg was used to generate cDNA using the SensiFAST^TM^ cDNA Synthesis Kit (Bioline #BIO-65053) according to manufacturer’s instructions. Gene expression analysis was conducted using the SensiFAST^TM^ SYBR^®^ Hi-ROX Kit (Bioline #BIO-92020) and detected using an Applied Biosystems 7900HT Fast Real-Time PCR system. The cycling conditions used were 95°C for 2 min, followed by 40 cycles of 95°C for 15 s and 60°C for 1 min, and a final melt curve analysis at 95°C for 15 s, 60°C for 15 s and 95°C for 15 s. Primers were used at a final concentration of 0.3 μM. The primers used were *Ppp2r1a* (forward 5′ ACTCTTCTGCATCAATGTGTT 3′, reverse 5′ ATACGAAGAACTGTGGGCA 3′), *Ppp2r1b* (forward 5′ GCTTCAGATGAACAGGACTCT 3′, reverse 5′ AGACAGT AACTGGGCAATGC 3′), *Ppp2ca* (forward 5′ CTTGTAGCTCT TAAGGTTCG 3′, reverse 5′ TCTGCTCTCATGATTCCCTC 3′), *Ppp2cb* (forward 5′ TTCTTGTAGCATTAAAGGTGCG 3′, reverse 5′ TCCATACTTCCGTAGGCAC 3′), *Ppp2r2a* (forward 5′ CCGTGGAGACATACCAGGTA 3′, reverse 5′ AACACTGT CAGACCCATTCC 3′), *Ppp2r2b* (forward 5′ GGACCTCAAC ATGGAAAATC 3′, reverse 5′ CGCTGTCTGACCCATTCCAT 3′), *Ppp2r2c* (forward 5′ AGCGGGAACCAGAGAGTAAG 3′, reverse 5′ GTAGTCAAACTCCGGCTCG 3′), *Ppp2r2d* (forward 5′ TTACGGCACTACGGGTTCCA 3′, reverse 5′ TTCGTCGT GGACTTGCTTCT 3′), *Rpl19* (forward 5′ CTCAGGAGATACC GGGAATCCAAGAAGA 3′, reverse 5′ CACATTCCCTTTGAC CTTCAG 3′). Genes of interest were normalized to the included housekeeping gene *Rpl19*, and relative gene expression was quantified using the comparative cycle (Ct) method (2^–ΔΔ*Ct*^) relative to wildtype.

### Histology and Immunohistochemistry

Embryos fixed in neutral buffered formalin (NBF) at various stages (E10.5, E14.5, and E18.5) were embedded in paraffin and sectioned at 5 μm before subsequent hematoxylin and eosin (H&E) staining or immunohistochemistry (IHC). Occasionally for H&E only, embryos were fixed in Bouins (Figure 4A E14.5 embryos). The H&E stained sections were analyzed by a veterinary anatomical pathologist at APN for stage-specific developmental features. IHC was performed as previously described (Pundavela et al., 2015). Briefly, following deparaffinization and rehydration steps using standard procedures, heat induced epitope retrieval (HIER) was carried out in a low pH, citrate-based antigen unmasking solution (Vector Laboratories, Burlingame, CA, United States, #H-3300) using a decloaking chamber (Biocare, West Midlands, United Kingdom) at 105°C for 5 min. After quenching of endogenous peroxidases in 0.3% H_2_O_2_, primary antibodies were prepared in 1% BSA, 0.1% Tween-20 in phosphate-buffered saline (PBS), and applied to the sections. Primary antibodies were rabbit polyclonal anti-cleaved caspase 3 (1:200; Cell Signaling Technology #9661), rabbit monoclonal anti-Ki67 [SP6] (1:100; Abcam, ab16667) and rabbit monoclonal p-cJun (1:100; Cell Signaling Technology #2361). The ImmPRESS (Peroxidase) Polymer secondary antibody kit and ImmPACT DAB (Vector Laboratories) were used for detection of the primary antibodies as per the manufacturer’s recommendations and the sections were counterstained with hematoxylin, dehydrated in a series of ethanol washes, and cleared in xylene before mounting with Ultramount No. 4 (Fronine #FNNII065C). Slides were scanned using a Leica Biosystems Aperio AT2 Scanner at 20× magnification.

### Immunofluorescence

Embryos were fixed overnight in 4% paraformaldehyde and cryopreserved through a sucrose gradient (10 and 30%) before freezing in O.C.T. Compound (Tissue-Tek^®^; Proscitech IA018) using isopentane cooled down in dry ice. Eight μm thick frozen sections were prepared using a Leica cryostat and immunofluorescence experiments were performed at room temperature, using a slightly modified procedure to previously described (Naudin et al., 2017). Briefly, sections were thawed, air dried, rehydrated in PBS and free aldehyde groups were blocked using a solution of 0.1M glycine in PBS for 10 min. Sections were then permeabilized in 0.3% Triton X-100 in PBS, and blocked with a solution of 1% BSA, 0.1% Tween-20 and 5% donkey serum in PBS for 30 min. Incubation with the primary antibodies was carried out at room temperature for 1 h before washing with PBS and applying the secondary antibodies for 1 h. The primary antibodies used were rabbit anti-keratin1 at 1:400 (Biolegend #Poly19052), rabbit anti-keratin14 at 1:400 (Biolegend #Poly19053), rabbit anti-loricrin at 1:500 (Biolegend #Poly19051), rabbit anti-B55α at 1:100 (Cell Signaling Technology #4953), an in-house rabbit polyclonal antibody against a PP2A-C peptide [PHVTRRTPDYFL (Sim et al., 1998)] at 1:500, a rabbit anti-Collagen IV at 1:100 (Millipore #AB756P), rat anti-Laminin γ1 at 1:1000 (Chemicon #1914), rat anti-Integrin β1 at 1:500 (BD Pharmingen #553715), rat anti-Integrin β4 at 1:200 (BD Biosciences #346-11A), rabbit anti-β-Catenin at 1:250 (Abcam # ab32572), and rabbit anti-ZO-1 at 1:100 (Thermo Fisher Scientific #61-7300). Alexa fluor-488 and -594 secondary antibodies (Abcam) were used at 1:500. All antibody dilutions were made in 1% BSA, 0.1% Tween-20 in PBS. After the secondary antibody incubation, slides were washed in PBS and mounted with Prolong gold antifade mountant with DAPI (Thermo Fisher Scientific #P36941), cured for 24 h in the dark at room temperature, coverslipped and stored at 4°C. Imaging was performed on a Fluoview FV1000 confocal microscope (Olympus).

### Keratinocyte Cultures

Primary keratinocyte cultures were derived as previously described by Li et al. (2017). Briefly, the skin from E18.5 embryos was collected, washed in PBS then incubated overnight at 4°C in 4 mg/mL ice cold dispase II (Merck #D4693) made up in Keratinocyte Serum Free Medium (ThermoFisher, #17005042) with added 1% Penicillin-Streptomycin antibiotic (Sigma #P4333). The next morning epidermis was isolated and incubated with the basal layer down on a 500 μL drop of 0.25% trypsin-EDTA in PBS with gentle rocking at room temperature for 20 min. Keratinocytes were mechanically detached by vigorously rubbing each epidermal sheet three times in 2 mL media. The detached keratinocyte suspensions were centrifuged at 180 × *g* for 5 min and seeded onto collagen I (Trevigen #3442-100-01) coated plates at a density of 1.67 × 10^5^ cells/mL. After growing to confluency, keratinocyte differentiation was induced by increasing the CaCl_2_ concentration in the media from 0.06 to 0.2 mM. Cells were imaged using a Zeiss Axiovert 200 inverted light microscope and Zeiss AxioVision software.

### Epidermal Barrier Staining

Immediately upon retrieval, E18.5 embryos were washed in PBS and incubated in X-gal reaction mix (100 mM NaPO_4_, 1.3 mM MgCl_2_, 3 mM K_3_Fe(CN)_6_, 3 mM K_4_Fe(CN)_6_ and 1 mg/mL X-Gal (Promega #V3941) at pH 4.5, with gentle rocking at 37°C for 3–4 h, followed by overnight incubation at room temperature. The following day, embryos were imaged using a digital camera.

## Results

### Constitutive Deletion of *Ppp2r2a* Is Lethal

**FIGURE 1:**
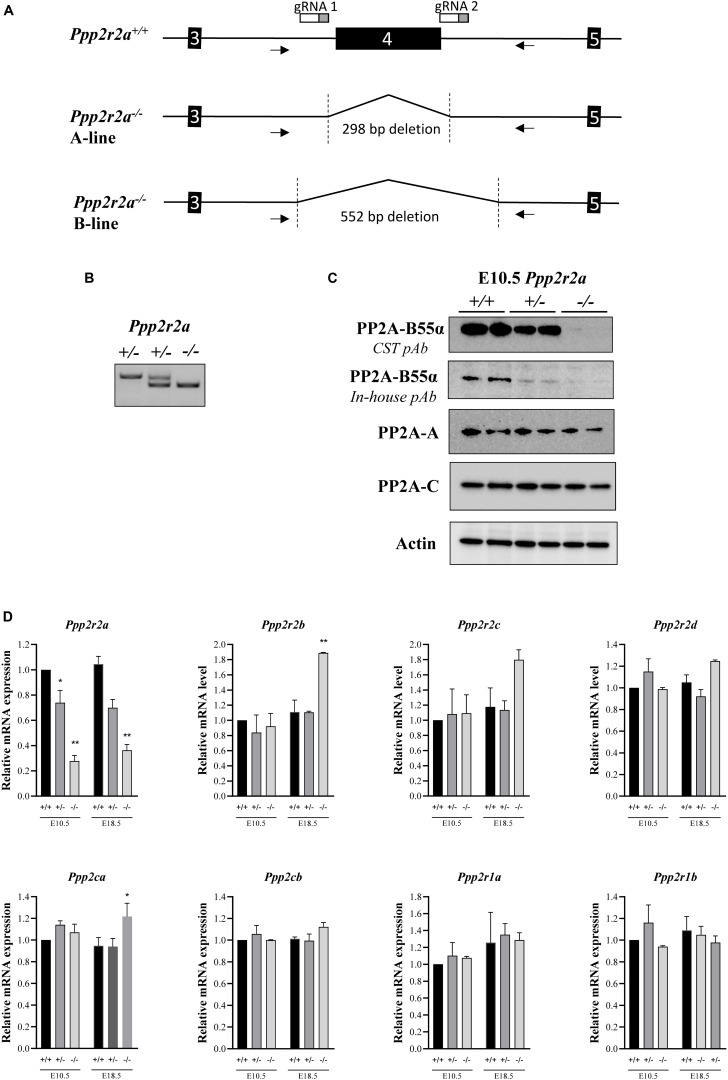
Generation of *Ppp2r2a* null mice lacking B55α protein expression. **(A)** Schematic of *Ppp2r2a* wildtype (+/+) allele and *Ppp2r2a* knockout alleles (–/–) from the A-line and B-line, showing the extent of deletion of exon 4 and partial flanking introns. The arrows show primer binding regions. gRNA 1 and 2 show binding site of CRISPR/Cas9 guide RNAs. **(B)** DNA genotyping gel of E10.5 embryos showing *Ppp2r2a* wild-type, heterozygous and knockout progeny. **(C)** Representative immunoblot of two E10.5 embryos of each genotype, revealing complete loss of PP2A-B55α expression in *Ppp2r2a* knockout (–/–) embryos using a primary polyclonal antibody to PP2A-B55α (CST #4953) and an in-house primary polyclonal antibody to B55α as described in the methods. Representative immuno-blots of PP2A-A and –C subunits. Actin was used as a loading control. **(D)** The mRNA expression of the B55 subunit genes (*Ppp2r2a, Ppp2r2b, Ppp2r2c, Ppp2r2d*), the PP2A catalytic subunit genes (*Ppp2ca* and *Ppp2cb)* and the PP2A structural subunit genes (*Ppp2r1a* and *Ppp2r1b*) were assessed by quantitative real time PCR. Data was normalized to the housekeeping gene, *Rpl19*, which was constant between genotypes and embryonic stages, and is represented as a fold change relative to the respective wildtype embryos for each stage. *n* = 2, *p < 0.05, **p < 0.01, two-tailed *t*-test compared to wildtype.

We generated *Ppp2r2a* knockout mice by targeting exon 4 using CRISPR/Cas9. Genotyping PCR primers flanking the targeted region and amplifying a product of 819 bp from the wild-type allele were used to characterize gene editing events in the resulting mice (Figure 1). Sequence analysis of the PCR products identified two mice with the expected deletion of exon 4 plus part of the flanking introns (Figure 1A and Supplementary Figure S1). These mice were used as founders to establish two independent *Ppp2r2a* knockout mouse lines, named *Ppp2r2a*-line A and B, respectively. The genomic deletion covers 298 bp in line A and 552 bp in line B (Supplementary Figure S1), removing *Ppp2r2a* exon 4 and resulting in a frameshift that prevents the expression of the full-length protein.

The founders were crossed twice to C57BL/6J before setting up heterozygous breeding pairs. In line A, out of 170 pups born from heterozygous breeding pairs, 100 (36%) were *Ppp2r2a*^+^*^/^*^+^, and 174 (64%) were *Ppp2r2a^+/–^* (Table 1). Similarly, in line B, out of 104 pups born from heterozygous breeding pairs, 43 (41%) were *Ppp2r2a*^+^*^/^*^+^, and 61 (59%) were *Ppp2r2a^+/–^* (Table 1). No homozygous knockout pups were observed at the time of genotyping (post-natal day 14) in either line, suggesting that constitutive deletion of *Ppp2r2a* results in early lethality with complete penetrance.

**TABLE 1:** Number of mice per genotype at various embryonic and post-natal stages.

	**Line A**					**Line B**
	**E10.5_a_**	**E14.5_b_**	**E18.5_c_**	**P0_d_**	**P14_e_**	**P14_f_**
*Ppp2r2a*^+/+^	14 (20%)	30 (29%)	30 (29%)	13 (37%)	100 (36%)	43 (41%)
*Ppp2r2a*^+/–^	38 (55%)	55 (55%)	62 (61%)	21 (60%)	174 (64%)	61 (59%)
*Ppp2r2a*^–/–^	17 (25%)	19 (18%)	10 (10%)	1^g^ (3%)	0 (0%)	0 (0%)

*a-8 litters; b-13 litters; c-17 litters; d-5 litters; e-70 litters; f-24 litters; g, this neonate was found dead the day of birth.*

Genotyping of embryos at E10.5 confirmed the presence of homozygous *Ppp2r2a^–/–^* embryos (Figure 1B) and immunoblot analysis using two different anti-B55α antibodies of whole E10.5 embryos revealed absence of B55α protein in *Ppp2r2a^–/–^* embryos, and reduced B55α protein in *Ppp2r2a^+/–^* embryos (Figure 1C). This was further corroborated by immunoblot analysis of E14.5 embryos (Supplementary Figure S2). There was no change in protein expression of the PP2A-C catalytic subunit, or the PP2A-A structural subunit.

Similarly, at the mRNA level, *Ppp2r2a* expression was decreased in *Ppp2r2a^+/–^* embryos and further reduced in *Ppp2r2a^–/–^* embryos compared to *Ppp2r2a*^+^*^/^*^+^ at both E10.5 and E18.5 (Figure 1D). This suggests that the altered mRNA synthesized from the *Ppp2r2a* knockout allele is unstable and mostly degraded, with only a small residual amount detected in *Ppp2r2a^–/–^* animals. We next assessed the expression of genes encoding the other B55 isoforms, and the catalytic and structural PP2A subunits. A significant increase in *Ppp2r2b* (B55β) expression was observed with *Ppp2r2a* knockout at E18.5. The expression of the *Ppp2ca* (encoding the PP2A-Cα subunit) was also increased with *Ppp2r2a* knockout at E18.5. The increased *Ppp2r2b* and *Ppp2ca* may be an attempt to compensate for the lack of B55α.

Heterozygous *Ppp2r2a^+/–^* mice in both the A and B lines grew normally, appeared healthy, were fertile and females nursed their pups. There were no major differences in size between *Ppp2r2a*^+^*^/^*^+^ and *Ppp2r2a^+/–^* mice monitored up to 24 months of age, and no obvious gross anatomical or behavioral problems were observed in *Ppp2r2a^+/–^* mice (Supplementary Figures S3A,B), despite a reduction in B55α protein expression observed in adult tissues (Supplementary Figure S3C). Further investigation into the *Ppp2r2a^–/–^* animals was focused on the A line.

### *Ppp2r2a* Is Required for Late Embryonic Development

The absence of homozygous *Ppp2r2a*^–/^*^–^* animals at 14 days of age suggested neonatal or *in utero* lethality. At E10.5, 25% of embryos harvested were *Ppp2r2a^–/–^*, consistent with the expected Mendelian ratio from a heterozygous cross. The percentage of *Ppp2r2a^–/–^* embryos at E14.5, however, dropped to 18% and it decreased further at E18.5, to only 10% (Table 1). At birth, this percentage dropped to 3%, corresponding to only one knockout offspring found amongst a total of 35 pups from 5 litters. It must be noted, however, that this neonate was found dead while litter mates were still being born, suggesting that it was still-born or died very soon after birth, indicating perinatal lethality.

### Late-Stage *Ppp2r2a*^–/–^ Embryos Are Small With Neural, Cranial and Limb Abnormalities

**FIGURE 2:**
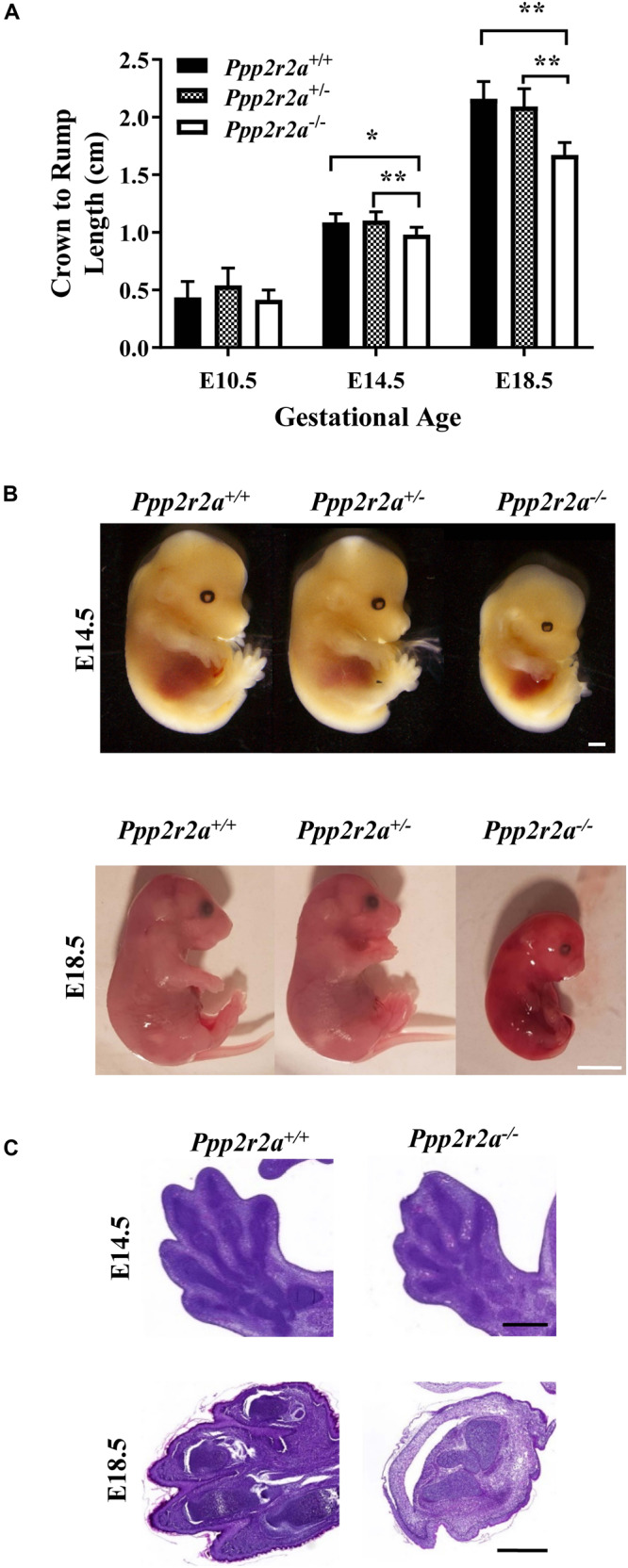
*Ppp2r2a* knockout embryos are small with epidermal hemorrhaging and syndactyly. **(A)** Crown-to-rump measurements of embryos at three developmental stages: embryonic day 10.5 (E10.5) (*n* = 5 per genotype), E14.5 (*n* = 7) and E18.5 (*n* = 7). *p < 0.05, **p < 0.01, unpaired two-tailed *t*-test. **(B)** Representative photographs of E14.5 and E18.5 embryos. *Ppp2r2a^–/–^* embryos display reduced size, syndactyly and gross epidermal hemorrhaging (E18.5). E14.5 scale bar = 0.5 mm; E18.5 scale bar = 5 mm **(C)**. H&E staining of E14.5 and E18.5 embryo sections demonstrates syndactyly of the hind limbs in *Ppp2r2a^–/–^* embryos. Scale bars = 0.5 mm.

To investigate the cause of the embryonic lethality, we first examined embryo size and gross morphology. At mid-gestation (E10.5), embryos of all three genotypes appeared macroscopically normal, and there was no difference in size, as determined by measuring the crown to rump length (Figure 2A). In contrast, *Ppp2r2a^–/–^* embryos at E14.5 and E18.5 were significantly smaller than *Ppp2r2a*^+^*^/^*^+^ and *Ppp2r2a^+/–^* embryos, and displayed abnormal morphology (Figures 2A,B and Supplementary Figure S4). Notably, a lack of divergent digits (syndactyly) was a common feature in all E14.5 (*n* = 19) and E18.5 (*n* = 10) *Ppp2r2a^–/–^* embryos, and was confirmed by histopathology analysis (Figure 2C).

**FIGURE 3:**
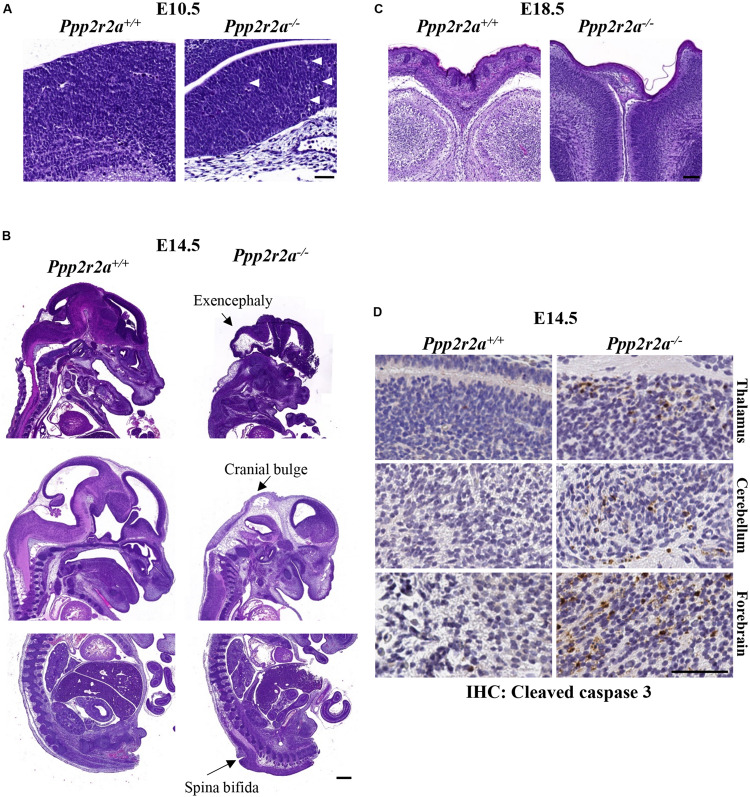
Neural defects in *Ppp2r2a* knockout embryos. **(A–C)** H&E staining at various stages of development. **(A)** At E10.5, defects of the neuroepithelium consist of a lack of emerging lamination and cellular degeneration and debris (white arrows) in *Ppp2r2a^–/–^*embryos. Scale bar = 50 μm. **(B)** At E14.5 the neural defects in *Ppp2r2a^–/–^* embryos include excencephaly, cranial bulging and spina bifida. Scale bar = 500 μm. **(C)** At E18.5, cranial vault collapse was observed in a *Ppp2r2a^–/–^* embryo. Also evident in these sections is a thinner epidermis in the *Ppp2r2a^–/–^* embryo. Scale bar = 100 μm. **(D)** IHC labeling for cleaved caspase 3 in the thalamus, cerebellum and forebrain of *Ppp2r2a*^+^*^/^*^+^ *and Ppp2r2a^–/–^* embryos. Scale bar = 50 μm.

While no macroscopic abnormalities were observed in E10.5 *Ppp2r2a^–/–^* embryos, histopathology analysis revealed differences in neural development. One litter analyzed displayed inconsistent neuro-epithelium in all three *Ppp2r2a^–/–^* embryos, compared to the one wild-type litter mate. Lamination of the cerebral cortex involves organization of the neurons into six layers in mammals (Chen et al., 2017). At E10.5 the *Ppp2r2a*^+^*^/^*^+^ embryo displays this emerging lamination (Figure 3A). This was not observed in the *Ppp2r2a^–/–^* embryos, but rather the neuro-epithelium displayed cellular degeneration and debris (Figure 3A). In contrast, in a second litter analyzed containing two *Ppp2r2a^–/–^* embryos, no histological differences were observed between the *Ppp2r2a*^+^*^/^*^+^ and *Ppp2r2a^–/–^* embryos (data not shown), suggesting incomplete penetrance of this phenotype at E10.5.

Out of 13 *Ppp2r2a^–/–^* embryos analyzed at E14.5, gross changes in cranial and/or embryo shape were observed in five embryos. Three embryos displayed a bulbous cranium, almost translucent compared to the rest of the body (Supplementary Figure S4A-representative). Another had a slight bulge at the cranial apex and small raised bulge along the upper dorsal region (Supplementary Figure S4B). The last also had a slight bulge at the cranial apex and a raised ridge-like structure along the dorsal region (Supplementary Figure S4C). These features suggested possible internal defects in the neural tube, such as exencephaly and spina bifida, and were confirmed by histopathology, with three *Ppp2r2a^–/–^* embryos displaying exencephaly (Figure 3B). One of these embryos also had cranial bulging due to edema at the cranial apex, and a protruding and poorly contained spinal cord in the cervical dorsal region (not shown). A further embryo was found to have spina bifida (Figure 3B).

Out of 10 *Ppp2r2a^–/–^* pups collected at E18.5, all showed movement or reacted to touch at the time of dissection. However, the skin of all E18.5 *Ppp2r2a*^–/^*^–^* embryos was shiny and displayed significant erythroderma, with visible superficial hemorrhaging particularly at the tips of the tail and limbs (Figure 2B). The morphology and histopathology of E18.5 *Ppp2r2a^–/–^* placentas (*n* = 3) appeared normal (data not shown). The only abnormal feature noted among the wild-type or heterozygous littermates was syndactyly in 1/30 (3.33%) *Ppp2r2a*^+^*^/^*^+^ embryos, however, this embryo was partially degenerated at the time of dissection and thus was likely a runt already undergoing the process of degradation. Histopathological analysis of four E18.5 *Ppp2r2a^–/–^* embryos found that one had cranial vault collapse (Figure 3C).

The one dead *Ppp2r2a*^–/^*^–^* pup observed at P0 (Table 1) was much smaller than *Ppp2r2a*^+/+^ and *Ppp2r2a^+/–^* littermates, and was partially degraded (Supplementary Figure S5). While *Ppp2r2a*^+/+^ and *Ppp2r2a^+/–^* pups were pale pink all over, the *Ppp2r2a*^–/^*^–^* pup was deep red in color, with the exception of a white tail, and exhibited an incomplete skin layer with large patches missing from the abdomen and a smaller patch on the lower spine, suggesting a potential epidermal barrier defect. As observed in the E18.5 embryos, the digits were also poorly defined.

We next investigated whether the cranial defects observed were associated with changes in aberrant cell proliferation or cell death. IHC staining for the proliferation marker Ki67, showed similar levels of Ki67^+^ cells in comparable brain regions of *Ppp2r2a*^+^*^/^*^+^ and *Ppp2r2a^–/–^* embryos at E14.5 (Supplementary Figure S6). There were very occasional cleaved caspase 3 (CC3) positive cells in the *Ppp2r2a*^+^*^/^*^+^ cerebellum and forebrain and none in the thalamus. In contrast, CC3^+^ cells were found in all three brain regions in the *Ppp2r2a^–/–^* brain (Figure 3D). This suggests the neuronal defects in *Ppp2r2a* knockout embryos are associated with increased apoptosis.

### *Ppp2r2a*^–/–^ Embryos Display a Thin Stratified Epidermis With a Defective Stratum Corneum

**FIGURE 4:**
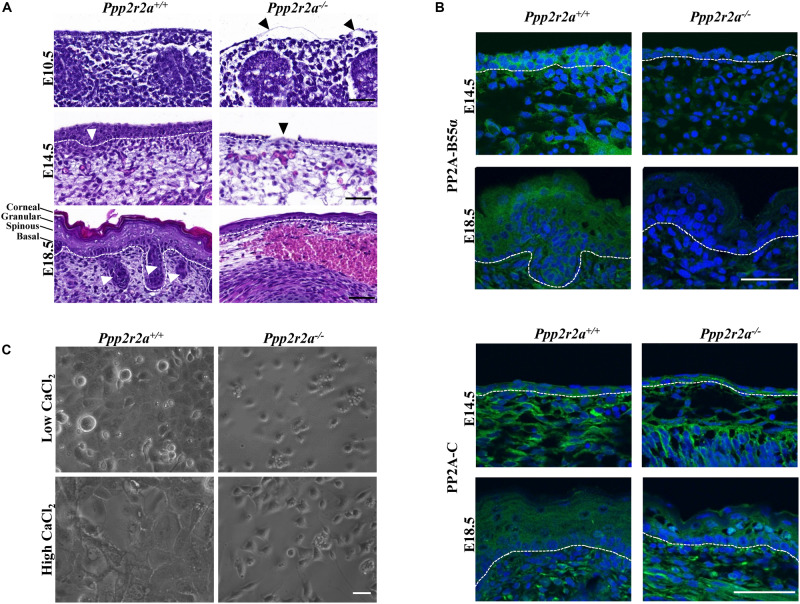
Epidermal defect in late stage *Ppp2r2a* knockout embryos. **(A)** Representative H&E images of the epidermis of E10.5, E14.5 and E18.5 *Ppp2r2a*^+^*^/^*^+^ and *Ppp2r2a^–/–^* mice. All three stages show thinner epidermal layers. E10.5 *Ppp2r2a^–/–^* embryos have regions of detached emerging epidermis (black arrowhead). Both E14.5 and E18.5 show subcutaneous hemorrhage and edema in the *Ppp2r2a^–/–^* embryos. *Ppp2r2a*^+^*^/^*^+^ display emerging hair follicles at E14.5, and dermal appendages at E18.5 (white arrowhead), but these are absent in *Ppp2r2a^–/–^* embryos. At E14.5 the *Ppp2r2a^–/–^* embryos also showed regions of disrupted epidermis (black arrowhead). Dashed white line indicates dermal-epidermal junction. Scale bars = 50 μm. **(B)** Representative immunofluorescence of PP2A-B55α (green) and PP2A-C (green) in *Ppp2r2a*^+^*^/^*^+^ and *Ppp2r2a^–/–^* epidermis at E14.5 and 18.5. Co-staining for DAPI is shown in blue. Dashed white line indicates the dermal-epidermal junction. Scale bars = 50 μm. **(C)** *Ppp2r2a*^+^*^/^*^+^ and *Ppp2r2a^–/–^* keratinocytes in low calcium and high calcium (post-48 h). Scale bar = 100 μm.

At E10.5, the epidermal committed basal layer in *Ppp2r2a^–/–^* embryos was much thinner and disrupted compared to the wild-type. In some instances, this layer was also detached from the underlying dermis (Figure 4A). As noted above, the skin of E18.5 *Ppp2r2a*^–/^*^–^* embryos was deep red with multiple sites of hemorrhaging. This was supported by histopathology analysis, with *Ppp2r2a*^–/^*^–^* embryos demonstrating superficial epidermal hemorrhage and edema at both E14.5 and E18.5 (Figure 4A). At E14.5, *Ppp2r2a*^–/^*^–^* embryos had a thinner basal epidermal layer than *Ppp2r2a*^+^*^/^*^+^ mice, with disorganized and irregular cell orientation, and a complete lack of an epidermal layer in some segments (Figure 4A, black arrowhead). Furthermore, they showed no emerging hair follicles – a hallmark feature of normal epidermis at E14.5 (Mann, 1962; Hardy, 1992; Cheng et al., 2014).

At E18.5, the epidermis of *Ppp2r2a*^–/^*^–^* embryos was also much thinner than *Ppp2r2a*^+^*^/^*^+^ embryos at the dorsal and caudal regions. The basal layer was often disorganized, the spinous and granular layers were thin, and the upper stratum corneum was less distinct and often missing altogether. These key phenotypic differences observed in the E14.5 and E18.5 epidermis were most prominent in the dorsal region. E18.5 *Ppp2r2a*^–/^*^–^* embryos also lacked dermal appendages, such as sweat glands, sebaceous glands and hair follicles, which are a normal feature of developing skin at this stage (Figure 4A; Hardy, 1992; Chu and Loomis, 2004; Schmidt-Ullrich and Paus, 2005).

Immunofluorescence analysis revealed PP2A-B55α expression in the basal and suprabasal layer of the epidermis, as well as dermal cells, in wild-type embryos at E14.5. At E18.5 PP2A-B55α was observed in all layers, with strongest expression in the granular layer (Figure 4B). The faint staining in the *Ppp2r2a*^–/^*^–^* embryos may be due to cross-reactivity of this antibody with the closely related B55δ subunit, however, lack of a specific B55δ antibody for immunofluorescence precludes us from confirming this. Immunofluorescence for PP2A-C showed a similar localization to PP2A-B55α at both embryonic stages (Figure 4B).

### *Ppp2r2a*^–/–^ Keratinocyte Cultures and Epidermis Display Impaired Differentiation

To further examine the epidermal phenotype of the *Ppp2r2a^–/–^* embryos, keratinocytes were isolated from the epidermis of E18.5 *Ppp2r2a*^+^*^/^*^+^ and *Ppp2r2a*^–/^*^–^* embryos. Interestingly, the epidermal layer separated more rapidly from the dermis of *Ppp2r2a^–/–^* than wild-type embryos, suggesting a weaker connection between the two layers. Less keratinocytes were consistently isolated from *Ppp2r2a^–/–^* skin, and these cells proliferated less than the wild-type cells (Figure 4C). Furthermore, the addition of high Ca^2+^ induced differentiation in the wild-type keratinocytes but not in knockouts. After 48 h in high Ca^2+^ the cytoplasm of *Ppp2r2a*^+/+^ keratinocytes expanded and the cells arranged in a distinctive cobble-stone pattern with clear cell-cell adhesions (Figure 4C). In contrast, the shape and pattern of the *Ppp2r2a*^–/^*^–^* keratinocytes showed very little change to untreated cells (Figure 4C), suggesting both proliferation and differentiation of isolated keratinocytes is impaired with PP2A-B55α loss.

**FIGURE 5:**
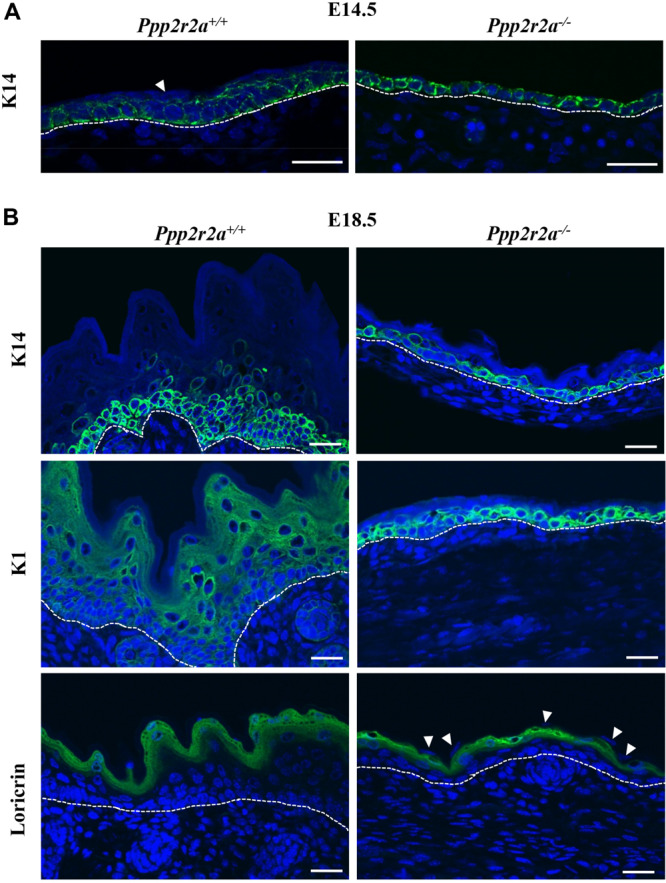
Epidermal defects are associated with abnormal differentiation. Immunofluorescent labeling for epidermal markers Keratin 14 (K14), Keratin 1 (K1) and Loricrin (green) in E14.5 **(A)** and E18.5 **(B)** embryos, with nuclei stained by DAPI (blue). White arrowheads show the unstained superficial epidermal cell layer. Dashed white line indicates the dermal-epidermal junction. All scale bars = 25 μm.

We next examined the epidermal proteins Keratin 14 (K14), a marker of proliferating basal cells; Keratin1 (K1), a marker of the spinous layer; and loricrin, a marker of the granular layer, by immunofluorescence labeling. At E14.5, K14 was present in both *Ppp2r2a*^–/^*^–^* and *Ppp2r2a*^+^*^/^*^+^ epidermis (Figure 5A). The *Ppp2r2a*^+^*^/^*^+^ epidermis was 1–2 cells thick, with all basal cells staining for K14, and the most prominent expression observed at the baso-lateral side of cells. In contrast, only one layer of cells was evident in the *Ppp2r2a*^–/^*^–^* epidermis, which was strongly positive for K14, with expression at both the apical and basal side, as well as the lateral junctions between cells (Figure 5A). Many cells in this single layer appeared flatter and irregularly positioned compared to the well-organized, regular cuboidal-type basal layer of the wild-type epidermis, indicating a potential defect in cell polarity in the *Ppp2r2a*^–/^*^–^* basal layer. In the *Ppp2r2a*^+^*^/^*^+^ epidermis, there was also a thinner layer of K14-negative cells (the intermediate layer) emerging above the K14-positive basal layers (Figure 5A indicated by a white arrow head), which is absent in the knockout epidermis.

At E18.5 in wild-type embryos all four layers of the epidermis have developed: basal, spinous, granular and stratum corneum (Figure 5B). Both the histological and immunofluorescence analysis showed that each layer of *Ppp2r2a*^–/^*^–^* epidermis was thinner than in the *Ppp2r2a*^+/+^ mice. K14 marked basal and spinous layer cells in wild-type E18.5 epidermis, however, similar to that observed at E14.5, only a single basal layer of K14^+^ cells was observed throughout most of the E18.5 *Ppp2r2a*^–/^*^–^* epidermis, and the cells were highly disorganized, further supporting a polarity defect (Figure 5B). K1^+^ cells were observed in spinous and granular layers of E18.5 *Ppp2r2a*^+^*^/^*^+^ epidermis. In contrast, strong K1 immunolabelling was observed in many cells within the basal layer of *Ppp2r2a*^–/^*^–^* epidermis. The overlying spinous layer was much thinner than the wild-type, and contained very few K1^+^ cells, and the very thin granular layer was negative for K1 (Figure 5B). Immunostaining for loricrin confirmed a thinner granular layer in the *Ppp2r2a*^–/^*^–^* epidermis (Figure 5B). Given cells in the stratum corneum are anuclear, the few unstained nuclei above the granular layer in the *Ppp2r2a*^–/^*^–^* epidermis (Figure 5B, indicated by white arrowheads) indicate a defective and persisting periderm.

### Reduced Basement Membrane and Integrins in *Ppp2r2a*^–/–^ Epidermis

**FIGURE 6:**
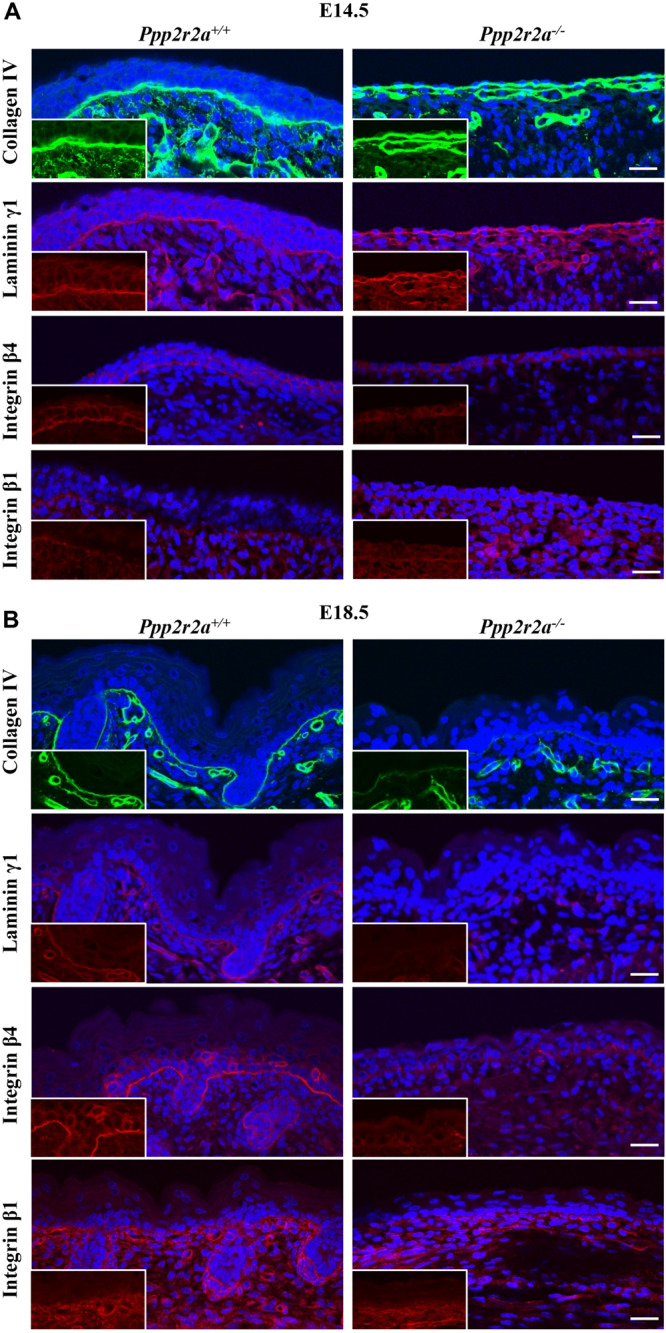
Reduced basement membrane and integrins in *Ppp2r2a* knockout epidermis. Representative immunofluorescent pictures of Collagen IV (green), Laminin γ1 (red), Integrin β4 (red) and Integrin β1 (red) in *Ppp2r2a*^+^*^/^*^+^ *and Ppp2r2a^–/–^* epidermis at both E14.5 **(A)** and E18.5 **(B)**. DAPI is shown in blue. Inserts show a region from each image without the DAPI to highlight the protein labeling. All scale bars = 25 μm.

Proper epithelial formation and integrity relies on formation of the basement membrane (BM), a specialized extracellular matrix (ECM) that is produced, secreted and assembled by the basal keratinocytes (Fuchs and Raghavan, 2002). The lack of mature stratified epithelium in the *Ppp2r2a* knockouts suggested there might be a defect in the BM. Immunolabelling for collagen IV and laminin γ1, two major components of the BM, was similar in both genotypes at E14.5 (Figure 6A), however, was markedly reduced in the *Ppp2r2a^–/–^* BM at E18.5 (Figure 6B).

The major cell surface receptors that recognize and assemble the BM are integrins; heterodimeric transmembrane proteins made up of an α and β subunit. Laminin binding to α3β1 integrin results in the assembly of focal adhesions, while binding to α6β4 integrin results in formation of hemidesmosomes, specialized adhesions that link the BM to the intermediate filament network (Tsuruta et al., 2008; Wickstrom et al., 2011). These integrin-mediated adhesions play critical roles in keratinocyte polarization and migration (Stepp, 1999; Rippa et al., 2013). At E14.5 β4 integrin was expressed on the basal side of the *Ppp2r2a*^+^*^/^*^+^ basal and suprabasal cells, whereas in the single layer of *Ppp2r2a*^–/^*^–^* cells, β4 integrin was expressed but did not show a preferential basal orientation. At E18.5, β4 integrin immunolabelling is strongest along the basement membrane of wildtype embryos. In contrast, in the *Ppp2r2a^–/–^* epidermis staining is weak and discontinuous (Figures 6A,B). Similarly, β1 integrin immunolabelling along the BM is weaker in the *Ppp2r2a^–/–^* dorsal skin, most notably at E18.5 (Figures 6A,B). The reduced BM and integrins would likely compromise the adhesion between the epidermis and dermis, and thus contribute to the blistering and ready separation of these layers described above.

### *Ppp2r2a*^–/–^ Embryos Have Aberrant Junctions in the Epidermis

**FIGURE 7:**
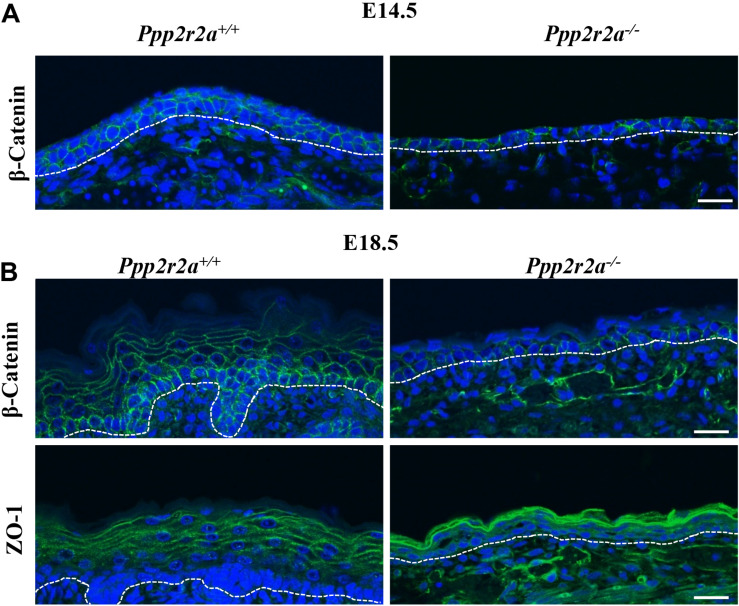
Aberrant cell-cell junctions in the *Ppp2r2a* knockout epidermis. **(A)** Representative immunofluorescent pictures of β-Catenin (green) labeling at E14.5 in *Ppp2r2a*^+^*^/^*^+^ *and Ppp2r2a^–/–^* epidermis. DAPI is shown in blue. **(B)** Representative immunofluorescent pictures of β-Catenin (green) and ZO-1 (green) in E18.5 *Ppp2r2a*^+^*^/^*^+^ *and Ppp2r2a^–/–^* epidermis. DAPI is shown in blue. Dashed white line indicates the dermal-epidermal junction. All scale bars = 25 μm.

In addition to the hemidesmosomes and focal adhesions attaching the epidermis to the BM, adherens and tight junctions play a key role in cell-cell attachment and formation of the protective epidermal barrier. β-catenin links membrane bound E-cadherin to the actin cytoskeleton at adherens junctions, and is also a key effector of the canonical Wnt pathway which is important in epidermal stratification (Capaldo and Macara, 2007; Hartsock and Nelson, 2008; Zhu et al., 2014). β-catenin labeling of the E14.5 *Ppp2r2a*^+^*^/^*^+^ epidermis is strongest along the cell-cell junctions between keratinocyte layers, and also between keratinocytes within a layer (Figure 7A). In contrast, β-catenin labeling in the *Ppp2r2a*^–/^*^–^* epidermis is inconsistent and patchy (Figure 7A). At E18.5, β-catenin is still highly expressed on the apico-lateral junctions between keratinocytes of the basal layer, and to a lesser extent in the spinous layers (Figure 7B). In comparison, weaker β-catenin is observed in the apico-lateral regions of the basal layer, and is absent from the thin suprabasal layers, in the *Ppp2r2a*^–/^*^–^* epidermis.

The tight junction protein, ZO-1 (Capaldo and Macara, 2007; Hartsock and Nelson, 2008) in E18.5 *Ppp2r2a*^+^*^/^*^+^ epidermis was localized at the cell-cell junctions of keratinocytes of the upper-spinous and granular layers (Figure 7B). Interestingly, ZO-1 junctional labeling was much stronger in the granular/spinous layers of *Ppp2r2a*^–/^*^–^* embryos compared to wildtypes but did not appear as well structured as seen on the wildtype (Figure 7B). Thus, the loss of PP2A-B55α affects the composition and/or organization of cell-ECM and cell-cell junctions in the developing skin.

### Late Stage *Ppp2r2a*^–/–^ Embryos Have Incomplete Epidermal Barrier Acquisition

**FIGURE 8:**
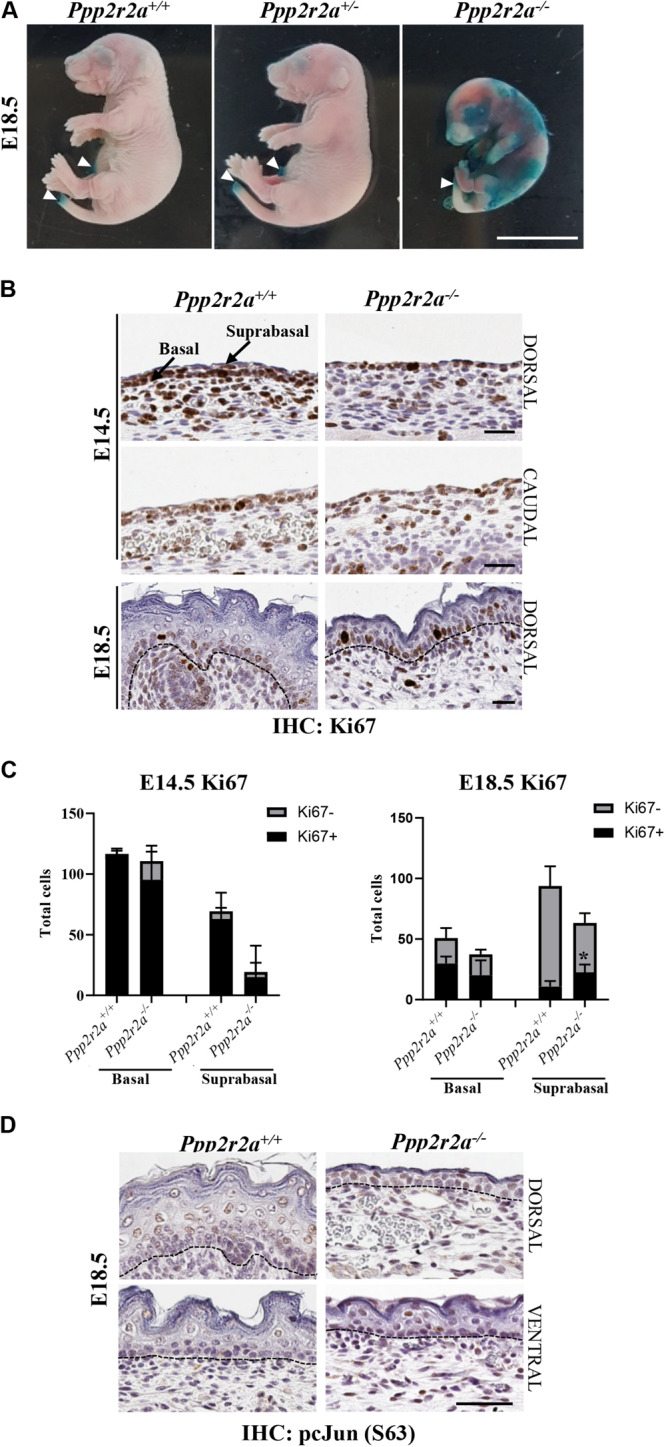
Defective epidermal barrier in *Ppp2r2a* knockout embryos is associated with changes in proliferation, and p-cJun signaling. **(A)** X-gal substrate penetration (permeability) assay in E18.5 embryos. Areas stained blue on the *Ppp2r2a^–/–^* embryo indicates regions with an impaired epidermal barrier. White arrowheads mark regions (umbilical cord and tail) that stained blue as they were cut during dissection. Representative of *n* = 3. Scale bar = 1 cm. **(B)** *Ppp2r2a*^+^*^/^*^+^ and *Ppp2r2a^–/–^* epidermal regions at E14.5 and E18.5 showing Ki67 (proliferation marker) staining. Dashed black line indicates the dermal-epidermal junction. **(C)** Quantitation of Ki67 positive and negative cells in E14.5 and E18.5 epidermis. Ki67^+^ cells were significantly increased in the *Ppp2r2a^–/–^* suprabasal epidermis compared to the wild-type. *p < 0.05, unpaired two-tailed *t*-test. **(D)** IHC for p-cJun (S63) in *Ppp2r2a*^+^*^/^*^+^ and *Ppp2r2a^–/–^* epidermal regions. Dashed black line indicates the dermal-epidermal junction. All scale bars = 50 μm.

The epidermis is essential for life as it provides a protective barrier, preventing water loss and protecting from external environmental factors (O’Shaughnessy et al., 2009). To test whether the epidermal abnormalities affected epidermal barrier function, a skin permeability assay was conducted on E18.5 embryos. In embryos with an incompletely formed epidermis the X-gal substrate will penetrate the skin, be cleaved by the endogenous β-galactosidase enzyme in the skin and form a blue precipitate in low pH conditions (Hardman et al., 1998; Guttormsen et al., 2008). In all *Ppp2r2a*^+^*^/^*^+^ and *Ppp2r2a^+/–^* embryos, only the edges of skin where the tail and umbilical cord were cut, turned slightly blue due to experimentally interrupted barrier. In contrast *Ppp2r2a^–/–^* embryos developed patchy blue staining all over the epidermis, confirming the presence of a disrupted epidermis, resulting in defective barrier function (Figure 8A).

### Altered Proliferation in *Ppp2r2a*^–/–^ Epidermis

The primary cultures suggested that proliferation may be impaired in the epidermis of *Ppp2r2a^–/–^* embryos. Therefore we stained for the proliferation marker, Ki67, via IHC. At E14.5, as noted above, the dorsal *Ppp2r2a^–/–^* epidermis was generally 1-cell thick, with only an occasional suprabasal layer, whereas the *Ppp2r2a*^+^*^/^*^+^ epidermis was 1–3 cells thick. However, in both genotypes the majority of epidermal cells were Ki67^+^ (Figures 8B,C). In E18.5 *Ppp2r2a*^+^*^/^*^+^ embryos, Ki67^+^ cells were present in the basal layer of the epidermis and surrounding the dermal appendages, with only a few positive cells in the spinous and subsequent outer stratified layers (Figures 8B,C). In comparison, the *Ppp2r2a^–/–^* epidermis contained fewer Ki67^+^ in the basal layer. In the suprabasal layer, where the cells are more compact in the knockout compared to the wild-type, there were reduced total numbers of cells, however, more cells were Ki67^+^. Therefore, homozygous *Ppp2r2a* deletion results in slightly reduced epidermal basal cell proliferation, but increased proliferation in the more apical suprabasal cells, which at this stage should be ceasing proliferation and starting to terminally differentiate.

### The Epidermal Defect in *Ppp2r2a^–/–^* Mice Is Associated With Increased Phosphorylation of the PP2A Target cJun

cJun is a subunit of the transcription factor AP-1, and functions in epidermal development downstream of PP2A and AKT (Karin, 1995; Kallunki et al., 1996). We found increased phosphorylation of cJun at S63, a site which enhances cJun transcriptional activity (Pulverer et al., 1991; Smeal et al., 1991; Deng and Karin, 1994), in the basal, spinous and granular layers of dorsal and ventral *Ppp2r2a^–/–^* epidermis, compared to wild-type epidermis (Figure 8D), suggesting loss of PP2A-B55α leads to hyperphosphorylation and activation of cJun in the developing skin.

## Discussion

We have generated and characterized the first constitutive *Ppp2r2a* knockout mouse. Homozygous deletion of *Ppp2r2a* resulted in embryonic lethality between E10.5 and birth, while heterozygous mice were viable and grew normally. Late stage *Ppp2r2a* knockout embryos were significantly smaller in size than littermate controls, and displayed various abnormalities such as syndactyly, neural defects and epidermal defects. The epidermal defect was characterized by thinner basal, spinous and granular layers, and a discontinuous stratum corneum. The structural defects of the epidermis were accompanied by a lack of dermal appendages and increased epidermal hemorrhage and edema. Functionally, the morphological skin defects translated to impaired epidermal barrier integrity, which is the likely cause of *Ppp2r2a*^–/^*^–^* neonatal death.

While this is the first reported *in vivo* analysis of *Ppp2r2a* deletion in mice, a study in zebrafish embryos revealed the importance of PP2A-B55α in cytoskeletal regulation to promote stable angiogenesis (Martin et al., 2013). Similarly, a prior *ex vivo* mouse embryo study had suggested that PP2A-B55α was essential in early embryogenesis (Liang et al., 2017). Double-stranded RNA-mediated *Ppp2r2a* knockdown in fertilized mouse zygotes arrested *in vitro* embryonic development, in association with increased DNA damage and apoptosis (Liang et al., 2017). In contrast, we observed close to Mendelian ratios of all three genotypes at E10.5, showing that PP2A-B55α is not essential for early embryonic development *in vivo*. However, the percentage of *Ppp2r2a^–/–^* embryos recovered at E14.5 and E18.5 steadily declined, suggesting that some knockout mice die between E10.5 and E14.5, others between E14.5 and E18.5, and a final few between E18.5 and P0. Therefore, there is significant heterogeneity in the penetrance of the phenotypes caused by *Ppp2r2a* loss.

The lethality observed in our *Ppp2r2a* knockout mice occurred later in embryogenesis than that reported in knockout mice for the PP2A structural and catalytic subunits (Götz et al., 1998; Ruediger et al., 2011). PP2A-Aα (*Ppp2r1a*) deletion resulted in the absence of homozygous knockout embryos by E10.5, but the cause of lethality was not determined (Ruediger et al., 2011). Given our E10.5 *Ppp2r2a* knockout embryos appeared grossly normal, we can infer that PP2A-B55α is not essential for implantation or gastrulation. However, the few occurrences of neural-related defects observed between E10.5 and E18.5 suggest that neurulation (∼E7.5) may not be fully functional. Homozygous PP2A-Cα knockout (*Ppp2ca^–/–^)* embryos had a developmental block at E6.5, before completing gastrulation (Götz et al., 1998). Moreover, *Ppp2ca^–/–^* embryos examined at E7.5 were found to be amorphous, smaller in size than controls and have a degenerated embryonic ectoderm (Götz et al., 1998). The early developmental block makes it difficult to compare the phenotype exactly to that of our *Ppp2r2a* knockout, however, some similarities are apparent; *Ppp2r2a* knockout embryos were also significantly smaller than wild-type littermates, and showed defects in neural and epidermal tissue, both of which derive from the ectodermal germ layer (Loebel et al., 2003). Thus, PP2A complexes containing B55α play an essential role in the development and differentiation of the ectoderm, but other PP2A regulatory subunit(s) are necessary, or are able to compensate for the loss of B55α, in the earliest stages of development.

Lamination refers to the differentiation of neurons into morphologically distinct layers in the neuroepithelium and begins around E10.0-10.5 (Chen et al., 2017). The emergence of these layers was seen in the E10.5 *Ppp2r2a*^+^*^/^*^+^ neuroepithelium (Figure 3A), but was not apparent in the *Ppp2r2a* knockout embryos. At E14.5 there is a continuation of neuroepithelium stratification and differentiation in wild-type mice, while at this stage *Ppp2r2a^–/–^* embryos exhibited neural tube defects. To our knowledge, PP2A has not previously been implicated in these developmental disorders. Exencephaly and spina bifida occur due to neurulation defects, such as inadequate neural tube folding, apposition or fusion, resulting in failure of neural fold closure (Harris and Juriloff, 2007; Chen et al., 2017). These defects begin early, with neurulation starting at ∼E7.5 and neural tube folding and fusion by ∼E10.5. The physical separation of the neuroectoderm from the surface ectoderm (future skin) also begins at ∼E10.5 (Chen et al., 2017). Thus, PP2A-B55α is important for normal development of the neuroepithelium.

There is a wide variety of genetic causes of neural tube defects [reviewed in Harris and Juriloff (2007, 2010)]. Mutation or reduced expression of numerous members of Wnt, FGF, or BMP pathways, for example FGFr1 and Axin, can lead to exencephaly and spina bifida (Juriloff and Harris, 2000). Neural cells develop from the ectoderm in the absence of canonical Wnt signaling when FGF signaling predominates and inhibits BMP. Conversely, an epidermal fate is adopted when active Wnt and BMP signaling predominates and promotes β-catenin activity (Finley et al., 1999; Stern, 2005; Fuchs, 2007). PP2A is known to regulate various proteins within these pathways and thus can have direct and indirect effects on neurulation. For example, it can positively regulate Raf-1, negatively regulate Erk downstream of FGF (Abraham et al., 2000; Jaumot and Hancock, 2001; Kubicek et al., 2002; Strack, 2002; Adams et al., 2005), and can exert both positive and negative regulation on Wnt/β-catenin signaling (Götz et al., 2000; Ivaska et al., 2002; Su et al., 2008; Clevers and Nusse, 2012; Mitra et al., 2012; Stamos and Weis, 2013; Thompson and Williams, 2018). PP2A-B55α complexes in particular can dephosphorylate β-catenin, leading to its stabilization and promotion of Wnt signaling (Zhang et al., 2009). Thus PP2A-B55α loss would be predicted to reduce Wnt signaling, and hence there may be no problem in committing to a neural fate, but rather in subsequent neurulation steps.

Further neural tube folding is promoted by the Sonic hedgehog (SHH) protein, alone and interacting with Wnts/BMPs (Colas and Schoenwolf, 2001; Ybot-Gonzalez et al., 2002). Loss of function of GLI3, a key SHH effector causes embryonic exencephaly, as does ectopic SHH expression (Echelard et al., 1993; Hui and Joyner, 1993). PP2A is also involved in SHH signaling via negative regulation of GLI3 (Krauß et al., 2008). AP-2 is a retinoic acid inducible transcription factor that is expressed by non-neuronal ectodermal cells but is also vital for neural tube closure, and AP-2 null mice develop exencephaly (Zhang et al., 1996). B55α containing PP2A complexes can dephosphorylate and decrease AP-2 activity (Ricotta et al., 2008). Similar pathways and proteins are also involved in syndactyly development, including the HOX genes (SHH and Indian hedgehog), FGFs, BMPs and Wnt, implying that PP2A-B55α regulates these pathways in multiple regions of the developing embryo (Manouvrier-Hanu et al., 1999).

The cellular degeneration and debris observed at E10.5 indicates that cell death is also occurring in *Ppp2r2a* knockouts. Whether this is due to PP2A specifically regulating cell death pathways, or is a consequence of the defective differentiation and the beginning of embryo degradation and resorption, is not clear. The neural defects observed at E14.5, were associated with increased apoptosis. Apoptosis is a normal and important part of shaping neuroepithelial development; however, it occurs in a very precise spatiotemporal manner. At later stages of gestation (E12–18), apoptosis occurs in regions that become the ventricular zone, intermediate zone, and the developing cortical plate and dorsal root ganglion of the cerebral cortex (Haydar et al., 2000; Yeo and Gautier, 2004; Chen et al., 2017). In the E14.5 *Ppp2r2a^–/–^* neuroepithelium, however, apoptosis was found in the thalamus, cerebellum and forebrain regions, which at this stage should be undergoing enlargement and differentiation (Chen et al., 2017).

*Ppp2r2a* deletion led to severe defects in epidermal stratification, including separation of the dermo-epidermal junction, subcutaneous hemorrhage and edema, and a thin and disorganized epidermis. Defective *Ppp2r2a^–/–^* keratinocyte differentiation was also evidenced in culture. These epithelial defects further indicate a failure of normal ectoderm differentiation with *Ppp2r2a* deletion. After neural tube fusion, the overlying epidermal ectoderm is separated from the neural tube and becomes what will be the skin at the back of the embryo (Colas and Schoenwolf, 2001). Interestingly, the most severe epidermal defects in the *Ppp2r2a* knockout occurred in the dorsal skin. Wnt/FGF/BMP signaling is important in induction of an epidermal fate and is also continually required through epidermal development and stratification (Zhu et al., 2014), providing further evidence for PP2A-B55α acting on these pathways in different regions of the developing embryo. In support of this hypothesis, B55α has been implicated in the regulation of BMP/TGF-β signaling by enhancing the TGF-β/Activin/Nodal signaling pathway (Batut et al., 2008). This pathway is essential for cell fate determination during development and for regulation of epidermal differentiation. Indeed, as mentioned previously, Wnt and BMP signaling are required for the ectoderm to develop into the epidermis, and this is further promoted by combined gradients of BMP and Nodal signaling during embryonic development which block neuroectoderm formation (Camus et al., 2006) and regulate patterning of the embryonic axes and neural and gut tubes (Arnold and Robertson, 2009; Pauklin and Vallier, 2015).

PP2A has previously been implicated in epidermal barrier formation. Mice with K14-Cre driven conditional knockout of PP2A-Cα had significant hair loss and disruption in the hair follicle regeneration cycle, as well as stunted size, melanin deposition and hyper-proliferation at the base of their claws (Fang et al., 2016). Furthermore, *ex vivo* studies have implicated the B55α subunit in epidermal development (O’Shaughnessy et al., 2009; Gerner et al., 2013; Youssef et al., 2013). Of the *Ppp2r2* family, the *Ppp2r2a* isoform is the most highly expressed during epidermal barrier acquisition in mouse embryos (O’Shaughnessy et al., 2009). Indeed, we found B55α protein to be expressed in all epidermal layers of E14.5 and E18.5 wild-type embryos. The epidermal defects observed with *Ppp2r2a* deletion now confirm an essential functional role for PP2A-B55α in epidermal development.

The formation of the epidermal barrier is a key element of late embryonic development that facilitates survival *ex-utero*, and should be completely formed by E18.5 in C57BL/6 embryos (O’Shaughnessy et al., 2009). However, the epidermal barrier in E18.5 *Ppp2r2a^–/–^* embryos was patchy and penetrable, which would inevitably result in the rapid death of pups at birth, as evidenced by the one *Ppp2r2a^–/–^* pup found dead at P0 that had visible skin defects. In the *Ppp2r2a^–/–^* epidermis the basal, spinous and granular layers were thin and poorly defined, and the stratum corneum was inconsistent or absent. The stratum corneum plays a major role in preventing water loss and protecting against the external environment (Sandilands et al., 2009). The presence of a few cells with intact nuclei in the uppermost layer of the *Ppp2r2a^–/–^* epidermis (above the loricrin stained granular layer Figure 5B) may indicate a persisting periderm, the transitory layer that normally disappears by ∼E17.5 (Sengel, 1976; M’Boneko and Merker, 1988; Zhang et al., 2005), and adds to the evidence of defective final cornification.

In the *Ppp2r2a^–/–^* epidermis there was also a lack of dermal appendages, which develop into hair follicles, sebaceous glands and sweat glands (Liu et al., 2013). Dermal appendages arise from interactive signaling between the epidermis and underlying dermis (mesoderm), and is mediated by similar signaling proteins and pathways as neural and epidermal development (Wnt, BMP, FGF, SHH) (Duverger and Morasso, 2009; Sennett and Rendl, 2012; Biggs and Mikkola, 2014; Ahn, 2015). Epidermal Wnt production modulates a BMP-FGF signaling cascade in the dermis and is essential for proper stratification and formation of the spinous layer, and for hair follicle initiation (Fu and Hsu, 2013; Zhu et al., 2014). This Wnt signaling cascade also promotes normal p63 expression in proliferating basal keratinocytes, and is required for the normal stratification process. When epidermal Wnt signaling is disrupted, the subsequent loss of p63 expression ablates the proliferative capacity of basal keratinocytes to properly stratify, resulting in a hypoplastic spinous layer, similar to the thin spinous layer of *Ppp2r2a* knockouts, thus further implicating aberrant Wnt signaling in lethality of these knockouts (Zhu et al., 2014).

Epidermal stratification and hair follicle formation also rely on β1-integrin mediated remodeling of the basement membrane. Epidermal specific (K14-Cre) β1-integrin knockout mice exhibit severe skin blistering and hair defects, accompanied by massive failure of BM assembly/organization, hemidesmosome instability, and a failure of hair follicle keratinocytes to remodel BM and invaginate into the dermis (Raghavan et al., 2000). Thus, the reduced β1-integrin in our *Ppp2r2a* knockouts likely contributes to the failure of developing hair follicles to invaginate into the underlying dermis.

The interaction and signaling between the epidermis and dermis via the BM, is also essential for keratinocyte polarization, proliferation and differentiation. α6β4 integrin heterodimers form hemidesmosomes by attaching to intracellular keratin filaments and anchoring basal keratinocytes to the BM (Jones et al., 1998). The BM layer was reduced and inconsistent in the *Ppp2r2a*^–/^*^–^* skin, and the expression of β4-integrin was also markedly reduced. β4-integrin null mice have hemidesmosome loss and show defective epidermal stratification, weak attachment to the basal lamina and basal and spinous keratinocyte disorganization leading to gross skin denuding (Dowling et al., 1996). Indeed, the separation of the dermis and epidermis, or blistering, and associated hemorrhage observed in the *Ppp2r2a*^–/^*^–^* skin (Figures 2B, 4A), are reminiscent of these β4-integrin knockouts. Furthermore, the stratified layers of these mice displayed mitotic basal-like keratinocytes, similar to the proliferative (Ki67^+^) cells observed in the suprabasal layers of the *Ppp2r2a^–/–^* embryos. Correct timing of hemidesmosome internalization and detachment from the BM is vital for formation of the spinous layer (Poumay et al., 1994). The ability of B55α null and β4-integrin null keratinocytes to maintain expression of basal cell markers and undergo mitosis indicates that loss of attachment to the BM and impaired polarization leads to premature release of basal cells into the stratified layers, without appropriate signals to terminally differentiate (Stepp, 1999; Rippa et al., 2013).

Keratinocyte polarity is not only vital for the appropriate cell-BM attachment, but also for correct orientation of the mitotic spindle, which under normal conditions is oriented parallel to the BM during basal layer formation, and changes to perpendicular to the BM to form suprabasal keratinocyte layers, with distinct differentiation markers (Smart, 1970; Lechler and Fuchs, 2005; Muroyama and Lechler, 2012). PP2A-B55α is known to play a role in cytoskeletal regulation and spindle dynamics during mitosis (Martin et al., 2013; Williams et al., 2014; Wang et al., 2015, 2018; Liang et al., 2017) thus, loss of PP2A-B55α-mediated regulation of cell polarity could cause defective “asymmetric” division, leading to accumulation of immature and mitotic suprabasal keratinocytes.

In addition to the reduction of β1- and β4-integrins, the *Ppp2r2a^–/–^* basal and spinous layers displayed reduced and inconsistent membrane labeling of the adherens junction associated protein, β-catenin. In contrast, the tight-junction associated ZO-1 labeling in the thin granular layers of *Ppp2r2a^–/–^* basal keratinocytes was stronger than the wildtype. Whether this is a compensatory mechanism to adjust for reduced hemidesmosomes, focal adhesions and adherens junction attachments remains to be determined.

The expression of K1 in *Ppp2r2a^–/–^* basal keratinocytes, which is normally restricted to keratinocytes committed to terminal differentiation (Sur et al., 2006), further indicates aberrant differentiation, and is reminiscent of ΔNp63 null mice (Romano et al., 2012). ΔNp63, a transcription factor with high homology to the tumor suppressor p53, and downstream mediator of Wnt signaling, is a master regulator of epithelial development and differentiation, and its deletion results in severe developmental abnormalities including truncated forelimbs, the absence of hind limbs, and a poorly developed stratified epidermis comprising isolated clusters of disorganized epithelial cells, with premature expression of markers associated with terminal differentiation, such as K1 (Romano et al., 2012). Like the *Ppp2r2a* knockouts, ΔNp63 null mice also had dramatic reduction in collagen and laminin BM proteins. PP2A was recently reported in ΔNp63 immunoprecipitates from squamous cell carcinoma cell lines (Katoh et al., 2016), but whether B55α directly or indirectly regulates ΔNp63 activity remains to be determined.

Taken together, our data suggests that PP2A-B55α is involved in epidermal development via regulation of WNT and BMP signaling pathways, together with cell polarity and adhesion regulation. Which specific substrates are involved in mediating these functions is not fully clear. cJun is a known target of PP2A-B55α (Gilan et al., 2015), and O’Shaughnessy et al. (2009) showed that there is a pulse of p-AKT leading to transient phospho-cJun (p-cJun) dephosphorylation during barrier acquisition (∼E17.5) and proposed that this occurs via PP2A-B55α. They further found that *Ppp2r2a* knockdown in skin explant cultures resulted in increased cJun phosphorylation and epidermal barrier incompetence (O’Shaughnessy et al., 2009). The increased p-cJun in the E18.5 *Ppp2r2a* knockout epidermis supports PP2A-B55α being the effector of AKT-mediated cJun dephosphorylation, and provides the first *in vivo* evidence for *Ppp2r2a* loss leading to defective epidermal barrier acquisition through increased aberrant p-cJun signaling, thus disrupting this spatiotemporally sensitive sequence of events that must occur at this time.

During the very late stages of embryonic and epidermal development, PP2A can also regulate the conversion of profilaggrin to free filaggrin monomers, which contribute to the protein scaffold upon which the uppermost impenetrable stratum corneum will attach (Kam et al., 1993; Sandilands et al., 2009). The lack of stratum corneum and dysfunctional epidermal barrier in the *Ppp2r2a* knockouts suggests that the PP2A-B55α regulatory subunit may regulate filaggrin. However, it is unlikely that PP2A-B55α regulation of cJun or filaggrin at these late stages of epidermal stratification are the only factors leading to the epidermal defects observed, particularly given defects were observed as early as E14.5. PP2A regulation of components of the Wnt, β-catenin, BMP, FGF, and SHH signaling pathways, are more likely to be driving neural development early on, and limb and epidermal development in later stages. Integrins, keratins, catenins, and tight junction proteins such as ZO-1, are all regulated by phosphorylation, therefore hyperphosphorylation of one or more of these proteins in the absence of B55α may also contribute.

In summary, our study provides the first *in vivo* evidence for the requirement of *Ppp2r2a* in embryonic development, with *Ppp2r2a* knockout causing limb, neural and epidermal defects. These aspects of embryonic development are for the majority controlled by key signaling pathways driving ectoderm development, such as Wnt/β-catenin, BMP, FGF and SHH. This highlights a novel role for PP2A-B55α in the control of ectodermal development. Future identification of the specific PP2A-B55α substrates mediating these effects will shed further light on the functional role of this essential protein phosphatase in normal development and disease.

## Data Availability Statement

All datasets generated for this study are included in the article/Supplementary Material.

## Ethics Statement

The animal study was reviewed and approved by the University of Newcastle Animal Care and Ethics Committee.

## Author Contributions

All authors contributed to this manuscript. NP, MC, CL-O, RK, and SR performed the experiments. NP, SR, and NV wrote the manuscript. NV and SR designed the study.

## Conflict of Interest

The authors declare that the research was conducted in the absence of any commercial or financial relationships that could be construed as a potential conflict of interest.
