# Supplementary material for: Ppp2r2a Knockout Mice Reveal That Protein Phosphatase 2A Regulatory Subunit, PP2A-B55α, Is an Essential Regulator of Neuronal and Epidermal Embryonic Development
Source: Front Cell Dev Biol. 2020 Jun 5;8:358. doi: 10.3389/fcell.2020.00358 (PMC7290052; doi:10.3389/fcell.2020.00358)
Supplement: Supplementary file 1 [file Data_Sheet_1.pdf]

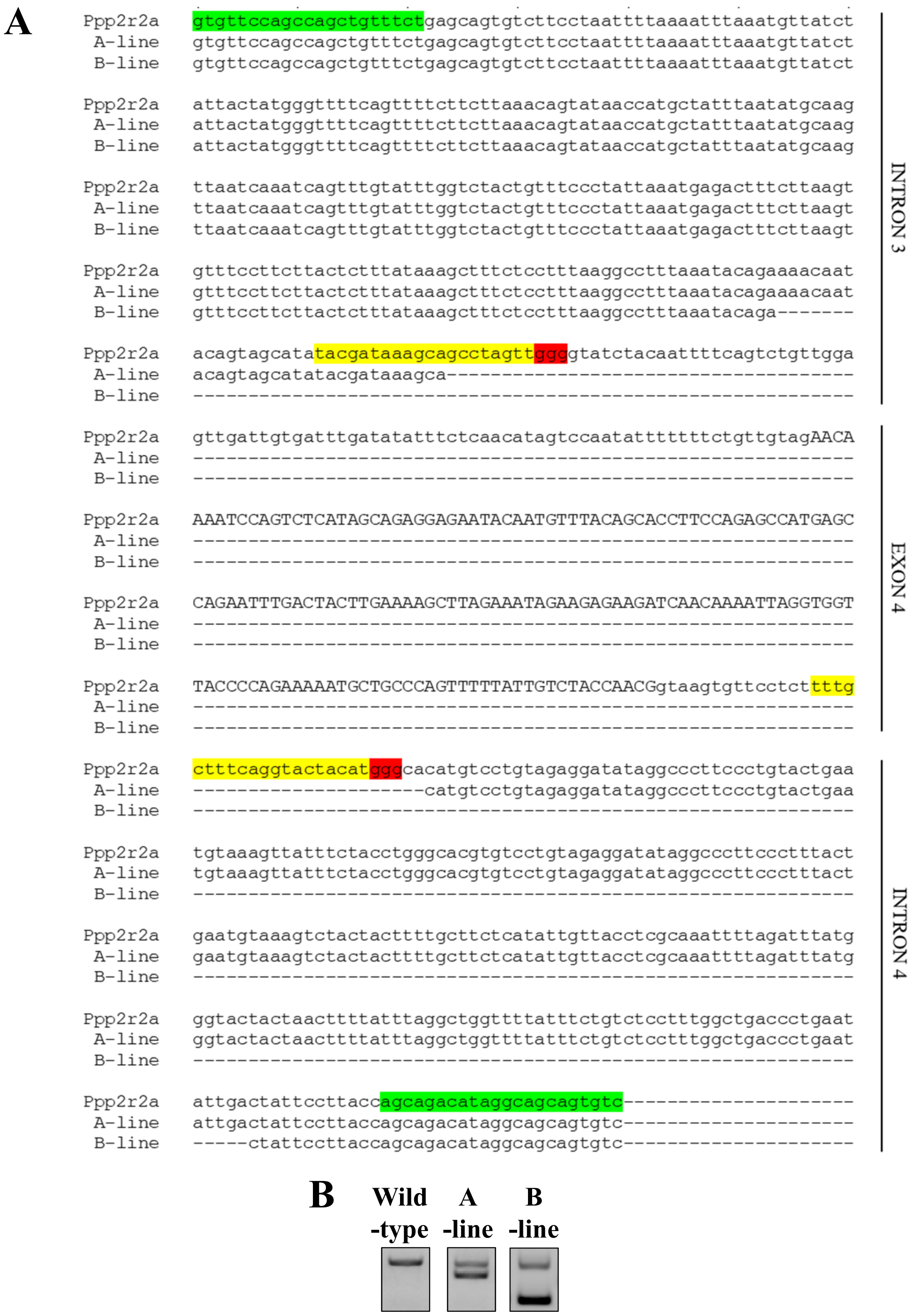


**Figure S1. *Ppp2r2a* wild-type sequence and deletion in knockout lines.**
**A.** Sequence alignment of wild-type *Ppp2r2a* exon 4 (upper-case) and flanking introns (lower-case) with the sequence of the A- and B- knockout lines. The alignment shows a deletion of 298 bp in the A-line and a larger deletion of 552 bp in the B-line. Exon 4 is completely deleted in both lines. Green shading highlights the binding of forward and reverse primers for PCR and subsequent sequencing. Yellow highlight shows the two gRNA sequences flanking exon 4, with red highlight marking the PAM sequence. **B.** DNA genotyping gels showing an 819 bp region amplified by primers in the wild-type allele in wild-type, *Ppp2r2a* line A and line B animals. Lower molecular weight bands in line A and line B correspond to the DNA area amplified in the knockout alleles. DNA gel of line B shows a smaller amplified area compared to line A due to a larger deletion in this line.

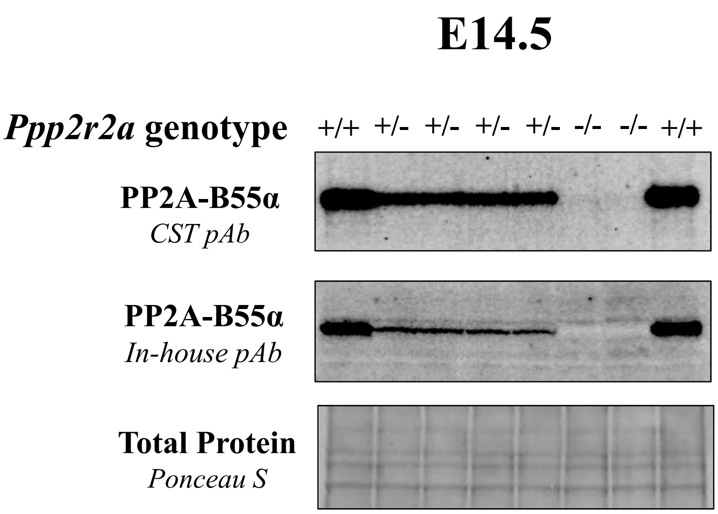


**Figure S2. PP2A-B55α protein expression in E14.5 embryos.** Western blot of B55α expression in the E14.5 embryos from *Ppp2r2a*$^{+/+}$, *Ppp2r2a*$^{+/-}$ and *Ppp2r2a*$^{-/-}$ mice. Immuno-blotting was performed as described in the methods, using two different polyclonal antibodies to PP2A-B55α (CST #4953, top; in-house, bottom) . Even loading was confirmed by ponceau S staining.

A

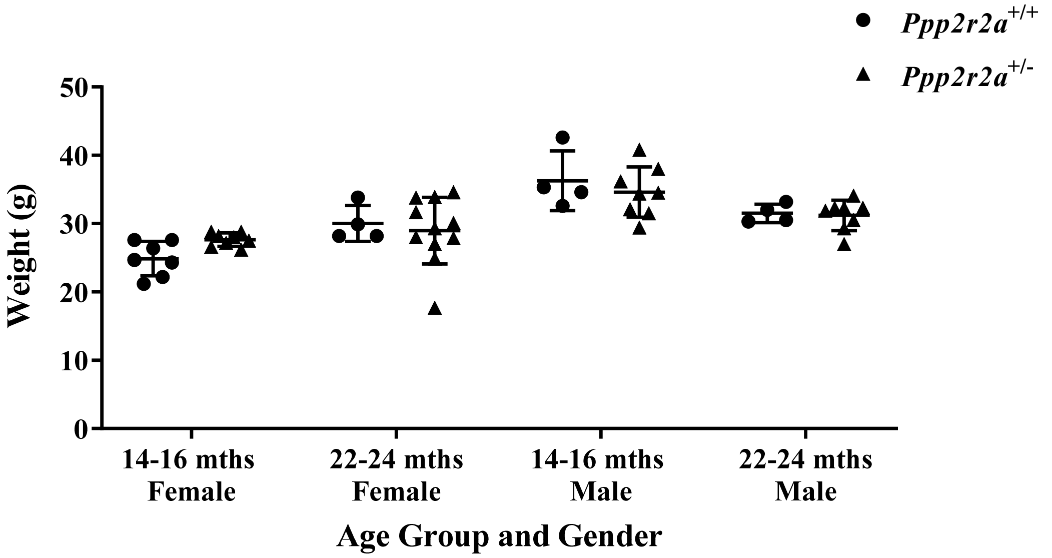


B

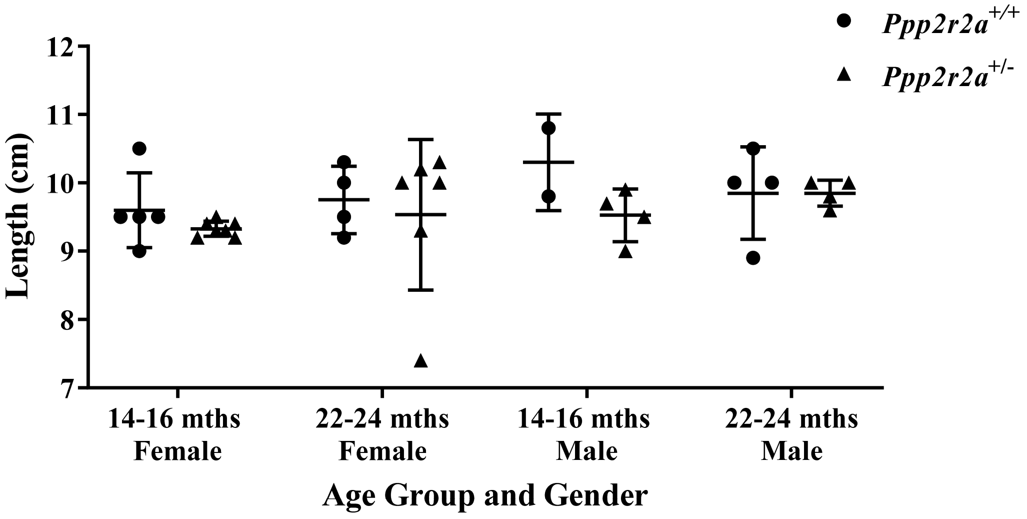


C

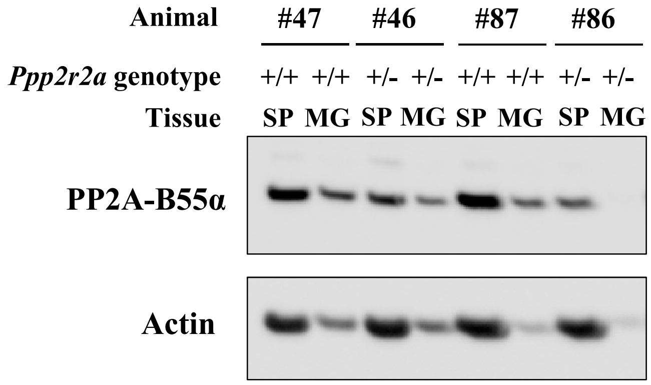


**Figure S3. Adult wild-type and heterozygous *Ppp2r2a* mice.** Weight (**A**) and size (**B**) differences between *Ppp2r2a*$^{+/+}$ and *Ppp2r2a*$^{+/-}$ mice of both genders aged up to 24 months. No significant changes were seen between genotypes in any groups. Unpaired two-tailed t-test. **C.** Western blot of PP2A-B55α expression in the spleen and mammary glands of 8-week old *Ppp2r2a*$^{+/+}$ and *Ppp2r2a*$^{+/-}$ mice. Protein from the tissues was extracted and solubilised using a mechanical tissue homogenizer (Ultra-Turrax) and RIPA lysis buffer. Immuno-blotting was performed as described in the methods, using a primary polyclonal antibody to PP2A-B55α (CST #4953) and a HRP-conjugated anti-actin antibody (Sigma #A3854) was used as a loading control.

**E14.5 *Ppp2r2a*$^{-/-}$ Gross morphological defects**

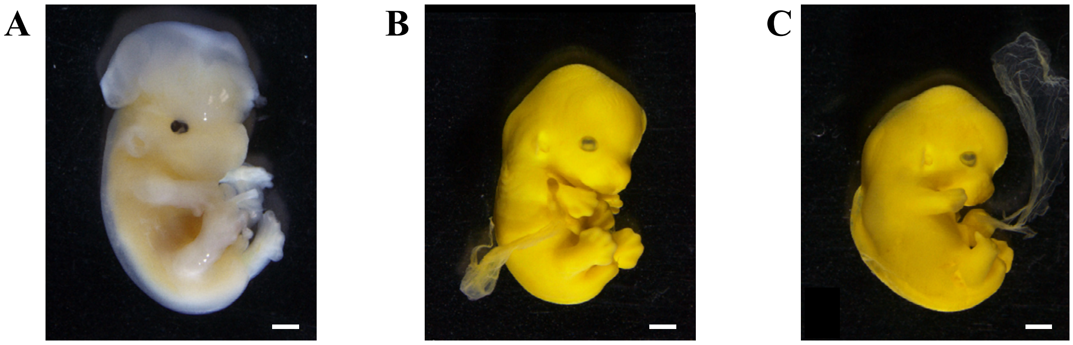


**Figure S4. Gross morphological defects in E14.5 knockout embryos.**
**A.** Embryo showing bulbous translucent cranium later confirmed as excencephaly via histology.
**B**. Embryo showing slight bulge at cranial apex and small raised bulge at cervical dorsal region.
**C.** Embryo showing slight bulge at cranial apex and a raised ridge-like structure along dorsal edge. Note that the embryo in A was fixed in 10% NBF and embryos in B and C were fixed in Bouins solution. Scale bars= 1000µm.

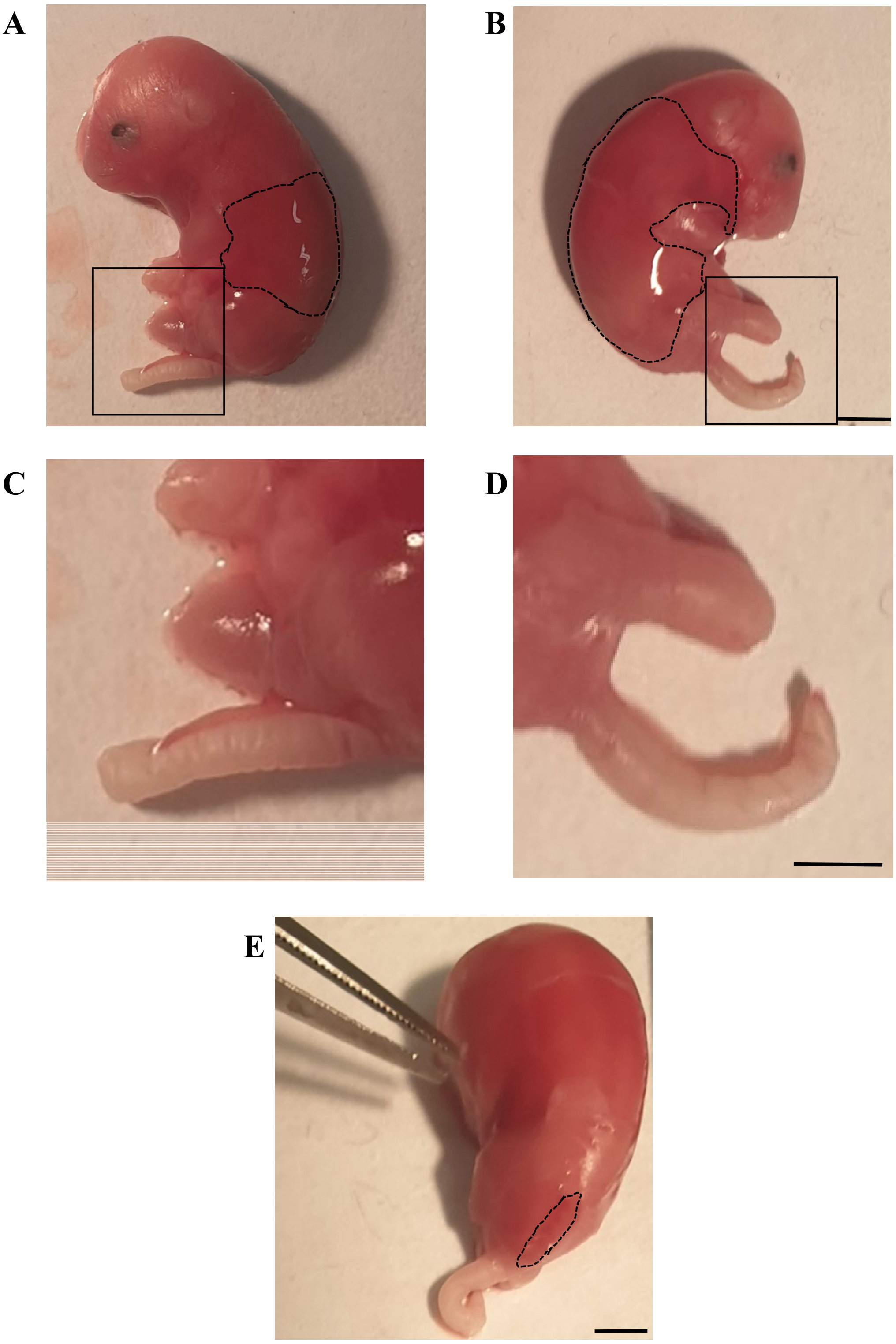


**Figure S5. Phenotype of dead knockout P0 pup.** Showing the left (**A**) and right (**B**) sides of the pup, with dashed lines indicating regions of missing skin (also notice the glossy morphology compared to matte of the areas with proper skin coverage). **C.** and **D.** show magnification of regions bound by rectangles in **A** and **B** respectively. Showing pale tail and undefined limbs and digits. **E.** shows the dorsal aspect of the pup, with an opening in the skin overlying the lower dorsal spine (marked by dashed lines). All scale bars= 2.5mm.

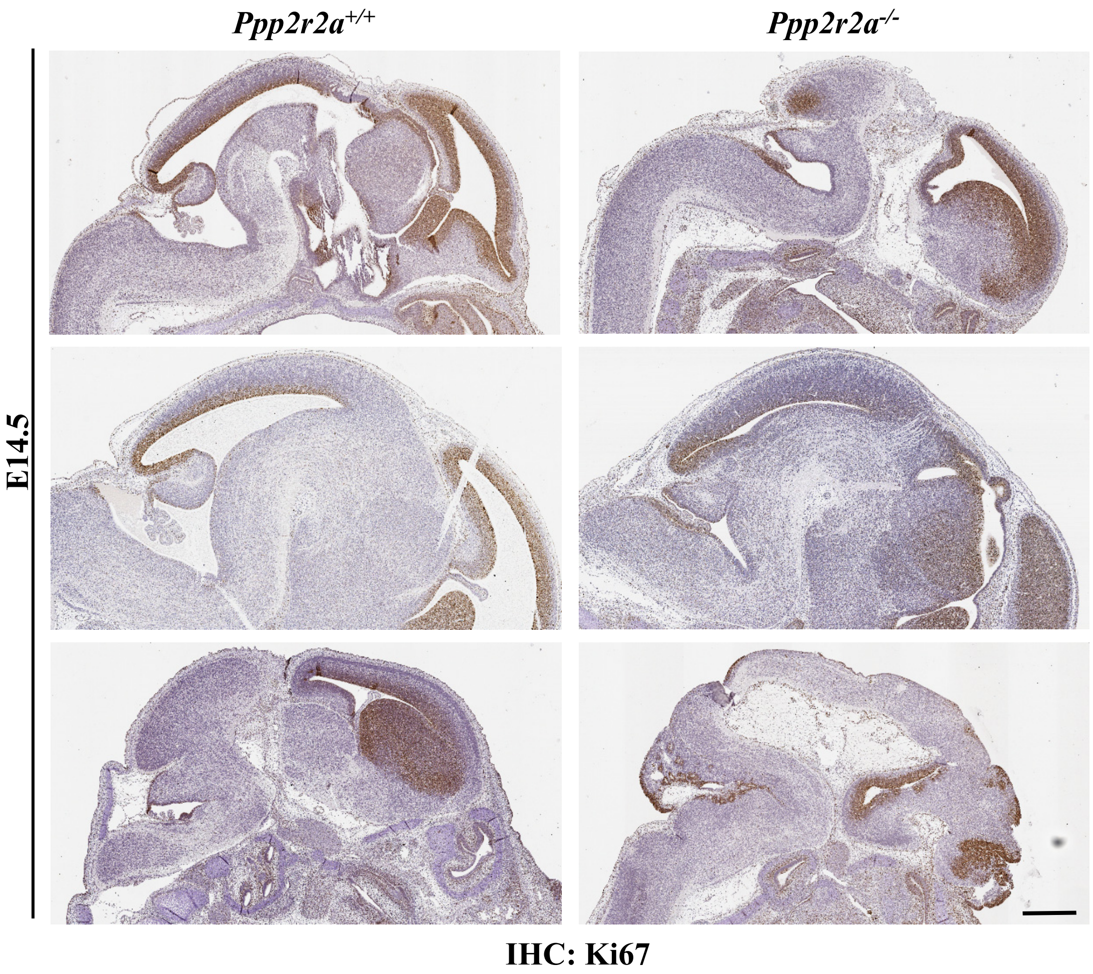


**Figure S6. Ki67 expression in the E14.5 embryo brain.** Ki67 expression via IHC in the brains of three *Ppp2r2a*$^{+/+}$ and *Ppp2r2a*$^{-/-}$ litter pairs, showing considerably similar staining in forebrain and midbrain regions, especially in cells surrounding the ventricles. The last *Ppp2r2a*$^{-/-}$ embryo brain has excencephaly which may account for different staining. Scale bar= 500µm.
